# Ultra-High Density, Transcript-Based Genetic Maps of Pepper Define Recombination in the Genome and Synteny Among Related Species

**DOI:** 10.1534/g3.115.020040

**Published:** 2015-09-08

**Authors:** Theresa Hill, Hamid Ashrafi, Sebastian Reyes Chin-Wo, Kevin Stoffel, Maria-Jose Truco, Alexander Kozik, Richard Michelmore, Allen Van Deynze

**Affiliations:** * Seed Biotechnology Center, University of California, Davis, California 95616; † Department of Plant Sciences, University of California, Davis, California 95616; ‡ Department of Horticultural Science, North Carolina State University, Raleigh, North Carolina 27695; § The Genome Center, University of California, Davis, California 95616

**Keywords:** GeneChip, Pseudolinkage, *Solanum tuberosum*, *Solanum lycopersicum* Solanaceae genomics

## Abstract

Our ability to assemble complex genomes and construct ultradense genetic maps now allows the determination of recombination rates, translocations, and the extent of genomic collinearity between populations, species, and genera. We developed two ultradense genetic linkage maps for pepper from single-position polymorphisms (SPPs) identified *de novo* with a 30,173 unigene pepper genotyping array. The *Capsicum frutescens* × *C. annuum* interspecific and the *C. annuum* intraspecific genetic maps were constructed comprising 16,167 and 3,878 unigene markers in 2108 and 783 genetic bins, respectively. Accuracies of marker groupings and orders are validated by the high degree of collinearity between the two maps. Marker density was sufficient to locate the chromosomal breakpoint resulting in the P1/P8 translocation between *C. frutescens* and *C. annuum* to a single bin. The two maps aligned to the pepper genome showed varying marker density along the chromosomes. There were extensive chromosomal regions with suppressed recombination and reduced intraspecific marker density. These regions corresponded to the pronounced nonrecombining pericentromeric regions in tomato, a related Solanaceous species. Similar to tomato, the extent of reduced recombination appears to be more pronounced in pepper than in other plant species. Alignment of maps with the tomato and potato genomes shows the presence of previously known translocations and a translocation event that was not observed in previous genetic maps of pepper.

Peppers, *Capsicum* spp., are, with few exceptions, diploid (2n = 2x = 24). The five domesticated species, *C. annuum* (*e.g.*, bell, Jalapeño, paprika, pimiento, chile), *C*. *frutescens* (*e.g.*, tabasco), *C*. *chinense* (*e.g.*, habanero), *C. pubescens*, and *C. baccatum*, are used as a spice to add pungency and flavor to salsas, curries, and dressings. Additionally, nonpungent, sweet *C. annuum* types are consumed as fresh or cooked vegetables, providing a rich source of Vitamins A, B, and C, iron, potassium, and magnesium. The diversity of uses for peppers has led to the development of individual types that have been selected for specific sets of consumer-driven fruit traits such as degree of pungency, flavor, color, shape, fruit wall thickness, and drying ability (Bosland and Votava 2000). Coincidently, peppers are bred for horticultural traits that allow them to be efficiently produced and withstand abiotic and biotic stress in temperate and tropical environments. World production of peppers has increased 32% from the years 2000 to 2010 (Fao 2012), valued at to $27.8 billion in 2011 (Fao 2012).

Pepper species are interfertile to varying degrees and are amenable to classical and molecular-genetic analyses. An integrated genetic map now includes more than 2200 mostly anonymous markers (AFLP, RFLP, SSR, and RAPDs) in 13 chromosomal linkage groups based on the integration of data from six intra- and interspecific populations (Paran *et al.* 2004). Doubled haploid and recombinant inbred line (RIL) populations increasingly have become available for mapping functional loci for disease resistance (*pvr*) and fruit quality (capsaicin, erect fruit habit, fruit color, fasciation, fruit shape) (Lefebvre *et al.* 2002; Ben Chaim *et al.* 2003; Ogundiwin *et al.* 2005; Barchi *et al.* 2007) as well as candidate gene analyses (Jeifetz *et al.* 2011; Brand *et al.* 2012; Cohen *et al.* 2012). Meta-analysis to identify quantitative trait loci (QTL) for *Phytophthora capsici* resistance leveraged these resources to refine the location of resistance loci (Mallard *et al.* 2013).

Genomic resources increasingly are becoming available in pepper and related Solanaceae, such as tomato (Tomato Genome Consortium 2012), potato (Potato Genome Sequencing Consortium 2011), and eggplant (Mueller *et al.* 2005; Hirakawa *et al.* 2014). Transcriptomes from multiple varieties and tissues have been sequenced and assembled for pepper as sequencing technologies advanced. For example, transcriptomes for fruit of two pepper parental lines (CM334 and Taean) and their hybrid (TF68) were sequenced with 454 technology (Lu *et al.* 2011, 2012). The Capsicum Transcriptome DB was created for *C. annuum* var. Sonora Anaheim and Serrano Tampiqueño with 32,314 contigs based on Sanger and 454 data (Góngora-Castillo *et al.* 2012). A composite assembly of normalized transcriptomes (from root, leaf, flower, and fruit tissues) of a *C. annuum* landrace, Criollo de Morelos 334 (CM334), Early Jalapeño, and a nonpungent blocky type, Maor, was generated with Illumina technology, totaling 123,261 contigs harboring 22,000 putative single-nucleotide polymorphisms defined at high stringency (Ashrafi *et al.* 2012). Transcriptome sequencing efforts also have facilitated the annotation of the recently assembled pepper genomes (Kim *et al.* 2014; Qin *et al.* 2014).

Ashrafi *et al.* (2012) also described the assembly of 31,196 unigenes from Sanger sequences that served as the basis for the design of a high-density Affymetrix microarray for pepper. This array was used to analyze polymorphisms in 40 *C. annuum* lines selected by commercial breeders worldwide as representing the primary breeding germplasm plus three additional species (Hill *et al.* 2013). Breeding germplasm was separated into four major clusters and landraces. An analysis of population structure showed that nonpungent types were the least diverse and derived from pungent types. In this paper, we describe the genetic mapping of these unigenes in one interspecific and one intraspecific mapping population, relating it to the draft genome sequence of pepper, and show synteny with other Solanaceae crop genomes.

## Materials and Methods

### Plant material

Two populations of RILs were used to construct genetic maps. The intraspecific NM06 population, provided by Dr. Paul Bosland (New Mexico University, Los Cruces, NM), consisted of 66 F_6_:F_7_ RILs and was derived from a *C. annuum* ‘Early Jalapeño’ × *C. annuum* ‘CM334’ cross (Sy *et al.* 2008). The interspecific FA07 population was developed by Dr. Molly Jahn at Cornell University (Ithaca, NY) and coauthors at UC Davis, was derived from a cross between *C. frutescens* accession BG2814-6 × *C. annuum* ‘NuMex RNaky’ and consisted of 123 F_7_:F_8_ RILs produced from single seed descent from F_2_ lines described by Ben-Chaim *et al.* (2006). We will refer to the NM06 and FA07 populations and maps as NM and FA, respectively.

### DNA extraction and polymorphism detection

DNA was extracted from each RIL and parent as described Hill *et al.* (2013). An Affymetrix GeneChip designed for detection of polymorphism in pepper was used for *de novo* identification of polymorphic markers segregating in each population. The pepper GeneChip contained sequences from the 31,196 unigene expressed sequence tag (EST) assembly (Ashrafi *et al.* 2012). After probe selection, 30,815 unigenes were represented on the GeneChip, including 642 nonpepper COSII gene sequences and 30,173 unigenes derived from pepper transcriptome and promoter sequences (Hill *et al.* 2013). Duplicate hybridizations of total genomic DNA were performed for each RIL, and three replicate hybridizations were carried out for each parent as described (Ashrafi *et al.* 2012; Truco *et al.* 2013). The raw hybridization data (CEL files) was subjected to robust multiarray average (Irizarry *et al.* 2003), background correction, and normalization and quality controls were as in Truco *et al.* (2013).

Polymorphic positions were detected within each RIL population as described previously for RIL genotyping with the similarly designed Lettuce and Pepper GeneChip arrays (West *et al.* 2006; Ashrafi *et al.* 2012; Hill *et al.* 2013; Truco *et al.* 2013). Briefly, weighted hybridization intensities of all probes spanning each 2-bp window for each unigene were used to calculate the SPPdev value at each 2-bp position for all the hybridized GeneChip arrays. The SPPdev ratio is a measure of the hybridization difference between modes of a bimodal distribution of SPPdev values for a given position across all samples. If no bimodal distribution was found, the position was considered nonpolymorphic. For each polymorphic position, an allele call (A, B, or −) was assigned for each GeneChip. This was carried out iteratively for all positions on all unigenes. A genotype was assigned to that 2-bp position based on the SPPdev values relative to the GeneChips hybridized with parental DNA; the “A” genotype was assigned to alleles from the cultivated parents *C. annuum* ‘Early Jalapeño’ (NM) and ‘NuMex RNaky’ (FA) and the “B” genotype to alleles from ‘CM334’ and *C. frutescens* acc. 2814-6. Both replicates of an individual RIL had to have the same genotype for an assignment to be made. If the replicates were inconsistent, the haplotype was treated as missing data. When contiguous 2-bp positions were polymorphic, they were summarized as one SPP range. Summarized SPPs were filtered on the basis of several criteria, including minimum average SPPdev value of 1.1, maximum percentage of missing data of a haplotype of 10%, minimum number of valid probes (those hybridizing to more than 90% of the antigenomic probes) of two, and minimum number of bases spanning an SPP of two. The number of SPPs per unigene ranged from 1 to 191, with averages of 7.1 and 9.9 SPPs per unigene for NM and FA populations, respectively. Consensus haplotypes were called from multiple SPPs within a contig.

### Linkage map construction

The haplotypes were determined for each RIL individual in the population with the methodology of Truco *et al.* (2013). Briefly, multiple SPPs within the same contig were summarized into consensus unigene haplotypes with a Python script (https://github.com/huaxudavis/xuhu-rwm-map). If the SPP calls within a unigene varied within a RIL, the two most likely consensus haplotypes were calculated and retained as markers for mapping (https://github.com/huaxudavis/xuhu-rwm-map/blob/master/split_spps.py). Four iterations of mapping were used to determine genetic bins and assign positions. For the first iteration, unigene markers were assigned to linkage groups based on consensus haplotype(s) using MadMapper (https://github.com/alex-kozik/atgc-map; https://github.com/alex-kozik/atgc-map/blob/master/Python_MadMapper_V254_RECBIT_V131010.py; https://github.com/alex-kozik/atgc-map/blob/master/py_matrix_2D_V254_RECBIT_V090710.py). Pairwise recombination values among all markers were calculated for each linkage group with JoinMap 4.0 (Van Ooijen 2006). Markers with zero recombination were considered to be one genetic bin. Therefore, within each line, the alleles for all markers within a bin should have originated from the same parent. The assignment of markers into genetic bins was not always unequivocal because of missing data. Adjacent markers had to be assigned to the same genetic bin when there was no recombination for some combinations of markers but there was recombination between other markers within the same bin (see Truco *et al.* 2013). Genetic bins consisted of single unigene markers (singletons), multiple unigene markers with the same haplotype, haplotypes differing only by missing data (marker bins), or haplotypes that having A, B, and missing calls for a given RIL where recombination could not be unambiguously determined (Truco *et al.* 2013). The marker with the least missing values within a genetic bin was used for mapping iteration one. Markers within a linkage group were ordered using RECORD (Van Os *et al.* 2005). CheckMatrix heat plots were used to visually inspect and adjust marker grouping and order. Genetic distances (in centimorgan) were then calculated with JoinMap 4.0 (Van Ooijen 2006). Based on bin map positions, all unigenes were ordered and graphical genotypes were inspected for obvious errors in unigene placement.

Because the SPP detection algorithm does not enable the determination of heterozygous alleles, mapping iterations two through four adjust for this by inputting heterozygous calls. Previous analyses have demonstrated that the SPP algorithm assigns heterozygous alleles as missing and A and B calls at a 2:1:1 ratio (Hill *et al.*, 2013). Regions of heterozygosity in individual RILs were identified by looking for contiguous regions with abnormally high rates of missing data compared with the same region in other RILs and high frequencies of apparent double crossovers. With the results of mapping iteration one, all SPPs were aligned with map positions. Graphical genotypes of the SPP calls were sorted by map position and visually inspected. SPPs with haplotypes inconsistent the bin haplotype, and other SPPs within their respective were removed. The missing data and allele frequencies across all remaining SPPs within each bin were then used to calculate the bin consensus allele for each RIL. Minimum requirements of <80% missing and 66% A or B among nonmissing calls were used to assign an allele. All other cases were considered missing. Homozygous haplotypes were distinguished from heterozygous haplotypes by the use of a sliding window of 5 cM. Heterozygous regions were defined as those where missing values, and multiple crossovers were detected over multiple contiguous 5-cM regions. For mapping iteration two, the alleles within the heterozygous regions were converted to missing values. Ordering and positioning of markers within each linkage group was determined with RECORD_WIN for iterations two through four. Consensus bin haplotypes were then recalculated for bins/unigenes that were collapsed into new bins by RECORD_WIN during iteration two. For iteration three, heterozygous regions were refined where the order of bins within heterozygous regions had changed after iteration two. The regions determined to be heterozygous were given heterozygous calls, not missing as in iteration two, then all remaining double crossovers were identified and changed to missing data and marker order and positions were calculated. Following iteration three, allele assignments by bin were again recalculated. Heterozygous calls that were not contiguous were refined and double crossovers supported by multiple SPPs at a 100% A or B frequency with no missing data were included for the final iteration with RECORD_WIN. All RECORD_WIN determined bins that showed not recombination were combined into the final genetic bins to represent each map.

For the FA map, linkage group assignment and orientation were based on conserved ortholog set II (COSII) markers between the parental lines *C. frutescens* var. BG2814-6 X *C. annuum* cv. NuMex RNaky that were previously assayed by Wu *et al.* (2009). Linkage group assignment for the NM map was based on markers common to the FA map. Because SPP detection parameters allowed a maximum allele frequency of 90%, skewed markers were included in the genetic maps. A χ^2^ goodness-of-fit test for 1:1 segregation of each bin marker was performed to identify significantly skewed genetic bins at *P* < 0.01.

Collinearity between maps was determined from common mapped unigenes by the assignment of rank order to the markers in each map. When there were multiple markers within a genetic bin, rank order within the bin was assigned on the basis of the order of those markers in the second map. A bivariate fit of rank order was performed and a linear fit model was calculated using JMP software (SAS Campus Drive, Cary, NC).

### Comparison of genetic maps to genome assemblies

The location of EST unigenes sequences in the pepper CM334 (v1.5) (Kim *et al.* 2014), pepper Zunla-1 (release 2.0) (Qin *et al.* 2014), tomato (SL2.50, http://solgenomics.net/organism/1/genome) (Fernandez-Pozo *et al.* 2015), and potato (Stuberosum_206_v3.4, http://genome.jgi.doe.gov/pages/dynamicOrganismDownload.jsf?organism=Stuberosum) (Grigoriev *et al.* 2012) genome assemblies were determined by the use of GMAP with default settings (Wu and Watanabe 2005). The BLAT output files were filtered to select high-confidence hits. The pepper genome matches were filtered at 98% identity, minimum alignment length of 200 nucleotides (nt), and a maximum of 50 unaligned nt at either end of the unigene. If there were multiple hits with the same score for a given EST after filtering, the position of the EST in the genome was considered ambiguous and dropped from further analyses. The tomato and potato genome hits were filtered at minimums of 80% identity and 75% coverage with a minimum length of 200 nt. When there were multiple hits for a given EST, the hit with greatest identity and coverage was retained. If there was no single hit with both greatest identity and coverage, the EST was excluded from further analyses.

Recombination rates (centimorgan per megabase pair) were calculated by taking the first derivative of the smoothed curve representing genetic *vs.* physical position (Ganal *et al.* 2011). The smoothed curve for each linkage group was calculated using the Kernal Smoother function in JMP.

### Data availability

The full set of SPP marker haplotypes, genetic maps (NM06 and FA07), comparison between maps, and mapping of unigenes to the Capsicum, Tomato, and Potato genomes have been added to the Sol Genomics Network FTP site (ftp://ftp.solgenomics.net/manuscripts/Hill_2015/). Additional information, including the GeneChip assembly, GO annotations for assembled unigenes, single-nucleotide polymorphisms, SSRs also is available (https://pepper.ucdavis.edu/public/data.php).

## Results

### Genotyping mapping populations using the Pepper GeneChip

The Pepper GeneChip genotyping array, containing ∼6.5 M 25-nt probes representing 30,815 unigenes, was used to detect polymorphisms within the intraspecific NM and interspecific FA RIL populations. The NM population was selected because it segregates for broad-spectrum resistance to *Phythopthora capsisi* from CM334 (Sy *et al.* 2008; Rehrig *et al.* 2014). The FA population was derived from the same cross producing F_2_ individuals previously used to generate a 299 marker COSII pepper map that was aligned with tomato linkage groups (Wu *et al.* 2009). Polymorphic positions (SPPs) within unigene sequences were detected on the basis of differential DNA hybridization values segregating within each population (Ashrafi *et al.* 2012; Hill *et al.* 2013). After polymorphism detection, three NM and four FA RILs were excluded from further analyses due to a high percentage of missing/ambiguous calls. Among the 63 NM and 119 FA RILs, 39,515 SPPs in 5587 unigenes (18% of those assayed) and 173,149 SPPs in 16,780 unigenes (55% of those assayed) were detected, respectively. Unigene consensus haplotypes (unigene markers) were used to generate genetic maps. The percentage of missing calls was 2.4% and 2.7% for NM and FA markers, respectively (Supporting Information, Table S1)

### Construction of genetic maps

**Figure 1:**
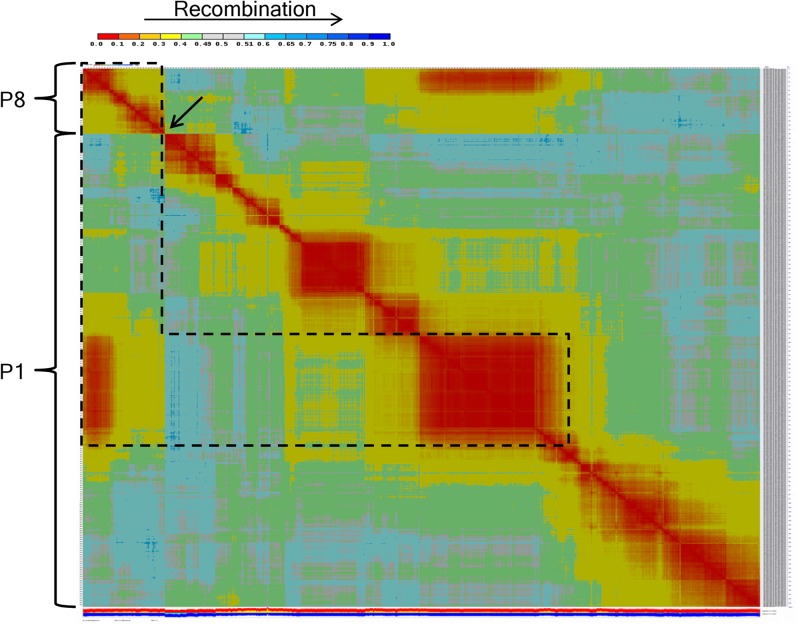
Heat plot showing recombination between markers. MadMapper cluster including FA linkage groups P1 and P8. The arrow indicates the position of a clear break in linkage between P8 markers (upper left) and P1 markers. The heat plot also indicates the pseudolinkage between P8 and P1 markers (black dashed line).

To construct the FA map, unigene markers clustered into 11 groups using MadMapper. Following marker ordering within clusters, visual inspection with CheckMatrix demonstrated that one cluster was interrupted by a sharp reduction in recombination indicating this cluster may include two linkage groups (Figure 1). The two groups clustered together due to linkage of markers in the middle of the large linkage block to those of the smaller linkage block. This pseudolinkage suggested that this cluster included the chromosomal translocation pair P1 and P8, as observed in previous interspecific maps (Tanksley *et al.* 1988; Livingstone *et al.* 1999; Wu *et al.* 2009; Park *et al.* 2014). This was confirmed by linkage group assignment that used FA COSII markers. A high proportion of FA markers, 96% (16,154 unigenes), were assigned to genetic bins (Table 1). There were 13 mapped FA unigenes having multiple haplotypes, and each of these mapped to two genetic bins. Each unigene with haplotypes mapping to two bins were considered as two distinct makers, resulting in a total of 16,167 markers in the FA map (Table 1 and File S1). The percentage of missing and heterozygous calls was 0.6% and 4.4%, respectively (Table S1). The FA map consisted of 2105 genetic bins with a total distance of 1380 cM. There were 62−303 genetic bins and 242−3137 markers per linkage group. The average and maximum intervals between genetic bins were 0.66 and 11.05 cM, respectively. To construct the NM map, 1452 NM markers were clustered by MadMapper into 21 groups. These 21 clusters were aligned with the FA linkage groups by the use of 1312 common markers resulting in 12 NM linkage groups. In addition to the 21 clusters, there were 115 unassociated markers. FA map markers were used to assign these markers to linkage groups where possible. Therefore, NM markers or NM marker clusters that did not have common FA map markers could not be assigned to linkage groups, and the markers were excluded from the map. The ordering of markers by RECORD at a critical gap size of 30 cM followed by visual inspection confirmed linkage group assignation with the use of a critical gap size of 30 cM. Linkage group assignment followed by marker ordering and calculation of genetic distances resulted in 3876 (69%) NM markers assigned to map positions. Two unigenes had markers that mapped to two genetic bins, resulting in a total of 3878 NM map markers (Table 1 and File S2). The NM map consisted of 783 genetic bins with a total genetic distance of the 1399 cM (Table 1). The average and maximum intervals between genetic bins were 1.81, and 10.48 cM, respectively (Figure S1). The number of genetic bins ranged from 53 to 102 and markers from 143 to 527 per linkage group. Linkage group P1 was the longest at 177 cM and P5 the shortest at 93 cM. The poorest marker coverage was found on P7, which had the fewest number of bins, total markers and the largest average marker interval.

**Table 1:** Statistics for *C. frutescens* acc. BG2814-6 × *C. annuum* ‘ NuMex RNaky’ (FA) and *C. annuum* ‘Early Jalapeño ’ × *C. annuum* ‘CM344’ (NM) maps

FA map						NM Map				
Linkage group	Bins*^a^*	Markers	Size, cM*^b^*	Bin Interval		Bins*^a^*	Markers	Size, cM*^b^*	Bin Interval	
				Maximum	Average*^c^*				Maximum	Average*^c^*
P1	303	3137	199	3.54	0.66	97	451	177	10.48	1.84
P2	183	1701	106	11.05	0.58	63	407	95	5.67	1.53
P3	244	2044	145	2.94	0.60	75	416	120	6.83	1.62
P4	186	1229	132	3.49	0.71	55	245	114	9.01	2.12
P5	151	968	99	5.95	0.66	52	246	93	7.7	1.83
P6	197	1435	136	3.81	0.69	70	283	138	7.71	2.00
P7	155	1226	107	2.58	0.70	49	143	115	8.72	2.40
P8	62	242	52	4.04	0.85	66	260	98	4.4	1.51
P9	148	986	116	7.02	0.79	63	527	100	7.83	1.61
P10	166	1078	101	1.88	0.61	71	310	128	8.7	1.83
P11	135	1001	91	2.50	0.68	62	363	98	5.97	1.61
P12	176	1120	96	2.14	0.55	60	227	123	8.54	2.08
Total	2105	16167	1380	11.05	0.66	783	3878	1399	10.48	1.81

a Unique map positions represented by unigene markers.

b Map length calculated based on unique markers representing each bin.

c Average marker interval (cM) = size/number of bins.

**Figure 2:**
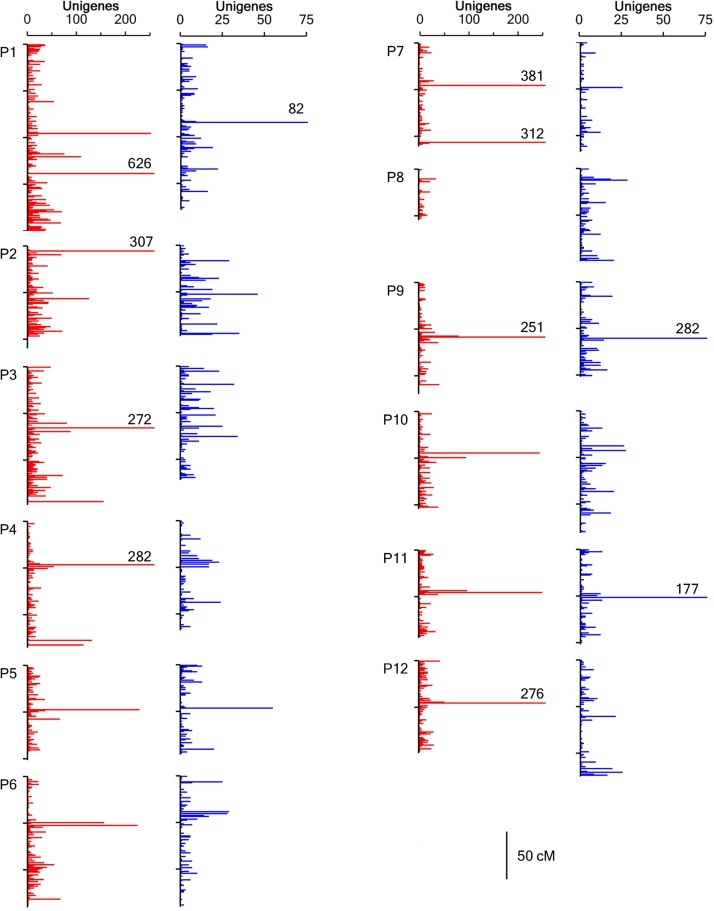
Marker density on FA (red) and NM (blue) linkage groups. Histograms show the marker density per centimorgan across all 12 Capsicum linkage groups. Bars represent the number of unigene markers and those labeled with values indicate the size of centimorgan bins greater than 200 (FA) or 75 (NM) markers.

**Figure 3:**
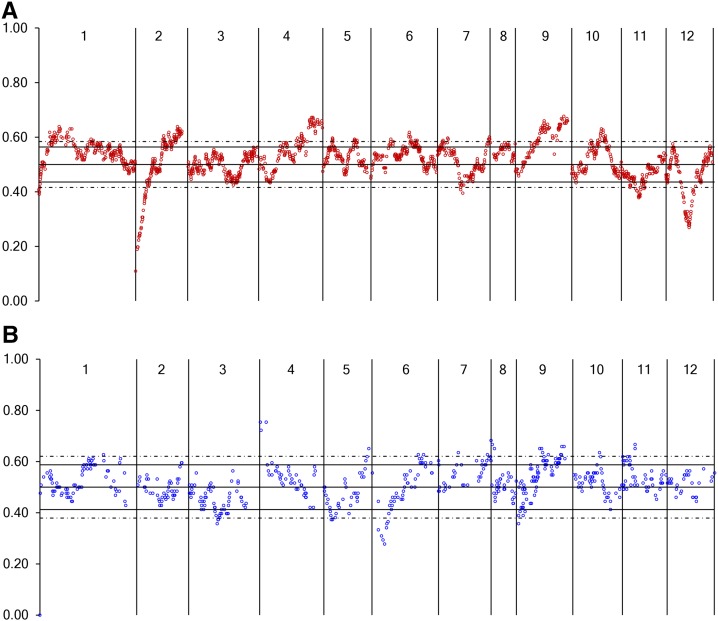
Allele frequencies of markers used to generate the genetic maps. Scatter plots show the allele frequency of each bin marker by genetic position for (A) the FA parent NuMex RNAKY and (B) the NM parent Early Jalapeño. Vertical lines delimit linkage groups indicated by the numbers at the top. The solid and dash horizontal lines represent allele frequencies resulting in significant χ^2^ values (*P* < 0.01 and *P* < 0.001, respectively).

Singletons accounted for 37% of both the NM and FA genetic bins (Table S2). Although there were few large gaps, greater than 5 cM and 2 cM between genetic bins for the NM and FA maps respectively, marker density per cM was highly variable (Figure S1 and Figure 2). Genetic bins having the largest numbers of markers were found on FA P1 at 137.5 cM with 624 unigene markers and NM P9 at 59.1 cM with 280 unigene markers (Table S3). Five linkage groups had 15% or greater difference in size between the FA and NM maps (Table 1). P3 (21%), P4 (15%), and P9 (16%) were longer in the FA map, and P10 (27%) and P12 (28%) were longer in the NM map. Markers with as low as 10% allele frequency were allowed during SPP detection and mapping. Markers having significant segregation distortion (*P* < 0.01) mapped to several linkage groups (Figure 3, Table S4, and Table S5). The NM map had distorted markers on nine linkage groups and seven distorted regions spanning more than 5 cM, four skewed toward the Early Jalapeño allele on P6, P7, P9 and P11 and three toward the CM334 allele on P3, P5, and P6. The FA map had skewed markers on 10 linkage groups, with 19 regions skewed toward the cultivated, NuMex RNaky, allele and six toward the *C. frutescens* allele. There were large regions (≥10 cM) distorted toward the NuMex RNaky allele on P1, P2, P10, and P11 and two regions on P4 and P9. The most highly skewed bins (*P* < 0.001) occurred across large regions at the tops of FA P2 (22 cM) and P12 (25 cM) toward the *C. frutescens* allele. Distorted markers were dispersed across different regions between the two maps with the exception of P9 where the pattern of distortion toward the cultivated allele appears similar between the two maps. Phenology and whole plant traits measured in the FA population by Yarnes *et al.* (2013) identified QTL for days to maturity, stigma exsertion, and branching density that lay within regions of skewed segregation (Table S5 and Table S6).

### Collinearity between FA and NM maps

**Figure 4:**
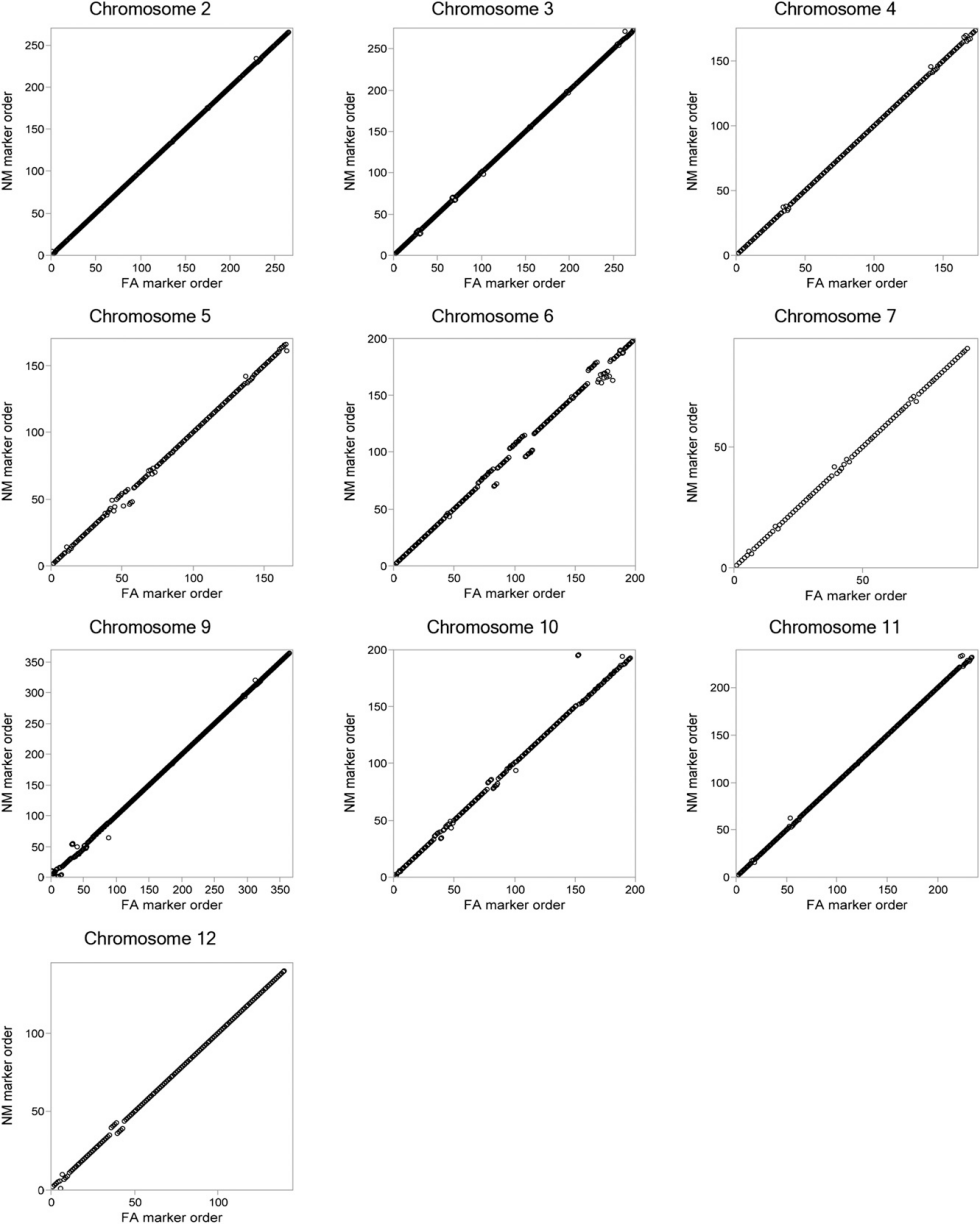
Regression of the order of markers on common linkage groups between the FA and NM linkage maps. The circles show the ordered 2108 markers common to both maps found on linkage groups 2–7 and 9–12 based on their map positions for both maps. Rank orders within each linkage group were used for regression analysis.

**Figure 5:**
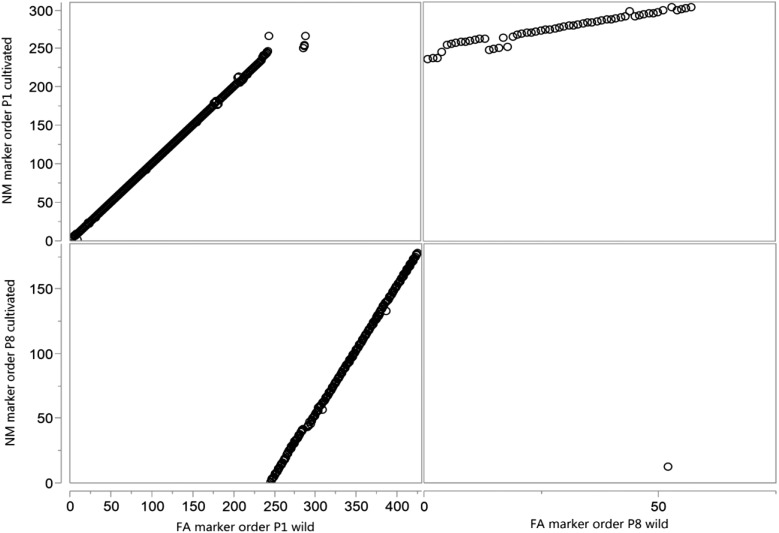
Regression of marker order between the FA (wild) and NM (cultivated) P1 and P8 linkage groups. The order of 483 markers common to both maps found on linkage groups P1 and P8, where a translocation has occurred between *C. frutescens* and *C. annuum*. Common markers were ranked based on their map positions for both maps then rank orders within each linkage group pair (P1 cultivated and P1 wild, P1 cultivated and P8 wild, P8 cultivated and P8 wild) were used for regression analysis.

There were a total of 2667 unigenes in common to both the NM and FA maps (Table S7). On the basis of on the relative genetic positions of the unigene markers on common linkage groups, there was a very high conservation of marker ordering between maps (Table 2, Figure 4, Figure 5, and Figure S2). The overall coefficient of collinearity among unigenes mapping to common linkage groups was 0.99, and collinearity coefficients by linkage group were 0.99−1.00, with the exception of the translocated distal arm of P1 cultivated and P8 wild at 0.96 (Figure 5). Minor obvious differences in collinearity between FA and NM maps were on P5, P6, P10, and P12 (Figure S2 and File S3).

**Table 2:** Collinearity between common mapped markers

Linkage Group	Common Markers	Coefficient of Colinearity*^a^*
P1 wild/P1 cultivated*^b^*	248	1.000
P8 wild/P1 cultivated*^c^*	56	0.963
P1 wild/P8 cultivated*^c^*	177	1.000
P8	1	−
P2	265	1.000
P3	274	1.000
P4	173	1.000
P5	166	0.999
P6	199	0.993
P7	91	1.000
P9	364	0.999
P10	196	0.993
P11	234	1.000
P12	140	0.999
Total same LG	2584	1.000
Other LG	83	
Total common	2667	

a Collinearity within each chromosome was assessed using common markers. The markers were ranked based on their map positions and the rank order was used for regression analysis, and expressed as R^2^.

b The top of P1 common to both *C. annuum* (P1 cultivated) and *C. frutescens* (P1 wild).

c Common markers between the bottom of P1 and P8. These chromosomes arms that have undergone a reciprocal translocation between *C. annuum* (cultivated) and *C. frutescens* (wild).

**Figure 6:**
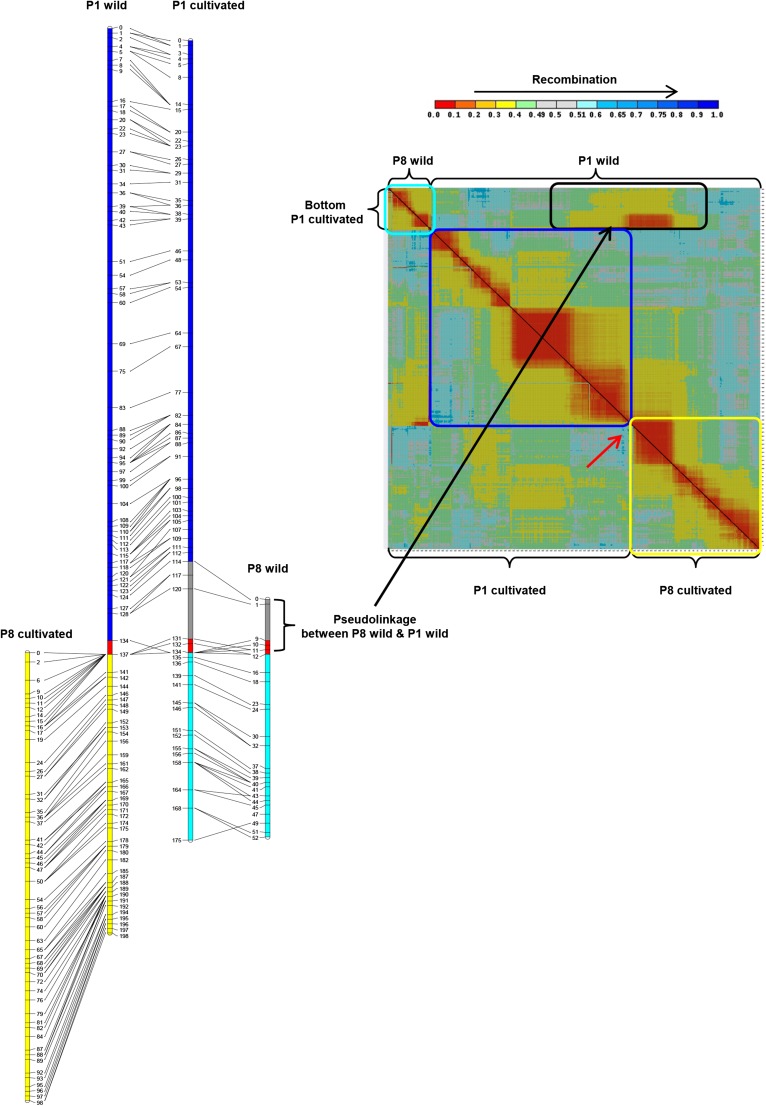
Recombination heat plot and painted chromosomes showing recombination between markers on P1 and P8 and common markers between FA and NM. The heat plot upper left triangle shows recombination between markers on FA P1 (wild) and P8 (wild). The lower right corresponds to NM P1 (cultivated) and P8 (cultivated) marker recombination. Chromosomal regions are colored according to translocation pairs. These regions are boxed in the same color on the heat plot. The nontranslocated portion of P1 (P1 wild/P1 cultivated) are shown in blue. The translocated arms P8 wild/P1 cultivated are in aqua and P1 wild/P8 cultivated are in yellow. Gray corresponds to the region of pseudolinkage between P1 and P8 wild and red indicates the region where the translocation break point occurred.

The large chromosomal translocation between the bottom arm of P1 and P8 between *C. annuum* and other *Capsicum* species was known to be segregating in the FA population resulting in P1 and P8 being represented by four chromosomes which have been designated P1 wild, P8 wild, P1 cultivated, and P8 cultivated (Tanksley *et al.* 1988; Livingstone *et al.* 1999; Wu *et al.* 2009). A comparison of P1 and P8 markers common to both maps demonstrated a clear break point on P1 wild (FA map) within the genetic bin at 137.5 cM (Figure 6). All common map markers distal to 137.5 cM on FA P1 wild map to P8 cultivated (NM map). Similarly, all common markers distal to NM 133.8 cM map to FA P8 wild. A number of markers mapping to P1 cultivated at 114.4−131.6 cM map to P8 wild at 0−12.1 cM in the FA population, however, several of these makers lack collinearity between the two maps indicating the association with P1 may be due to pseudolinkage between P1 wild and P8 wild markers.

Only 83 unigenes mapped to different linkage groups between populations, excluding P1/P8 (Table S7). When queried against the Illumina transcriptome assembly of Ashrafi *et al.* (2012) and the Zunla and CM334 genome assemblies (Kim *et al.* 2014; Qin *et al.* 2014), 77 and 40 of these unigenes had multiple transcriptome and genome hits, respectively. In addition, sequences of 22 unigenes were found on both chromosome pseudomolecules to which they were mapped. This finding suggests, that unigenes mapping to different linkage groups between populations tend to belong to multigene families and are mapping to different paralogs. In total, 76 of the 83 unigenes were found on a chromosome pseudomolecule in common with at least one of the mapped linkage groups with more matches to FA, 62 unigenes, than NM, 38 unigenes, linkage groups.

### Collinearity between genetic and physical positions

**Figure 7:**
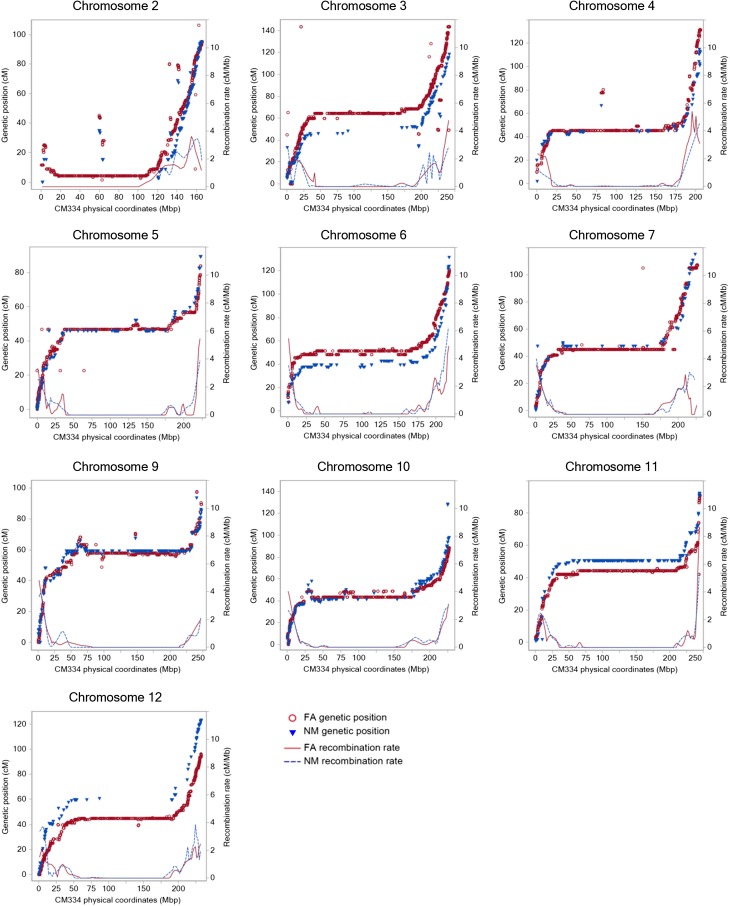
Relationship between physical and genetic positions with corresponding recombination rates. The 8825 FA and 2131 NM map markers that have been placed on chromosomes 2–7 and 9–12. The left Y-axis represents the centimorgan position of FA (red circles) and NM (blue triangles) unigene markers and the X-axis represents the physical position (Mbp) of unigenes on the CM334 pseudomolecules. The right Y-axis represents the recombination frequency, cM/Mbp, along the chromosomes for the FA (red line) and NM (blue dash line) maps.

**Figure 8:**
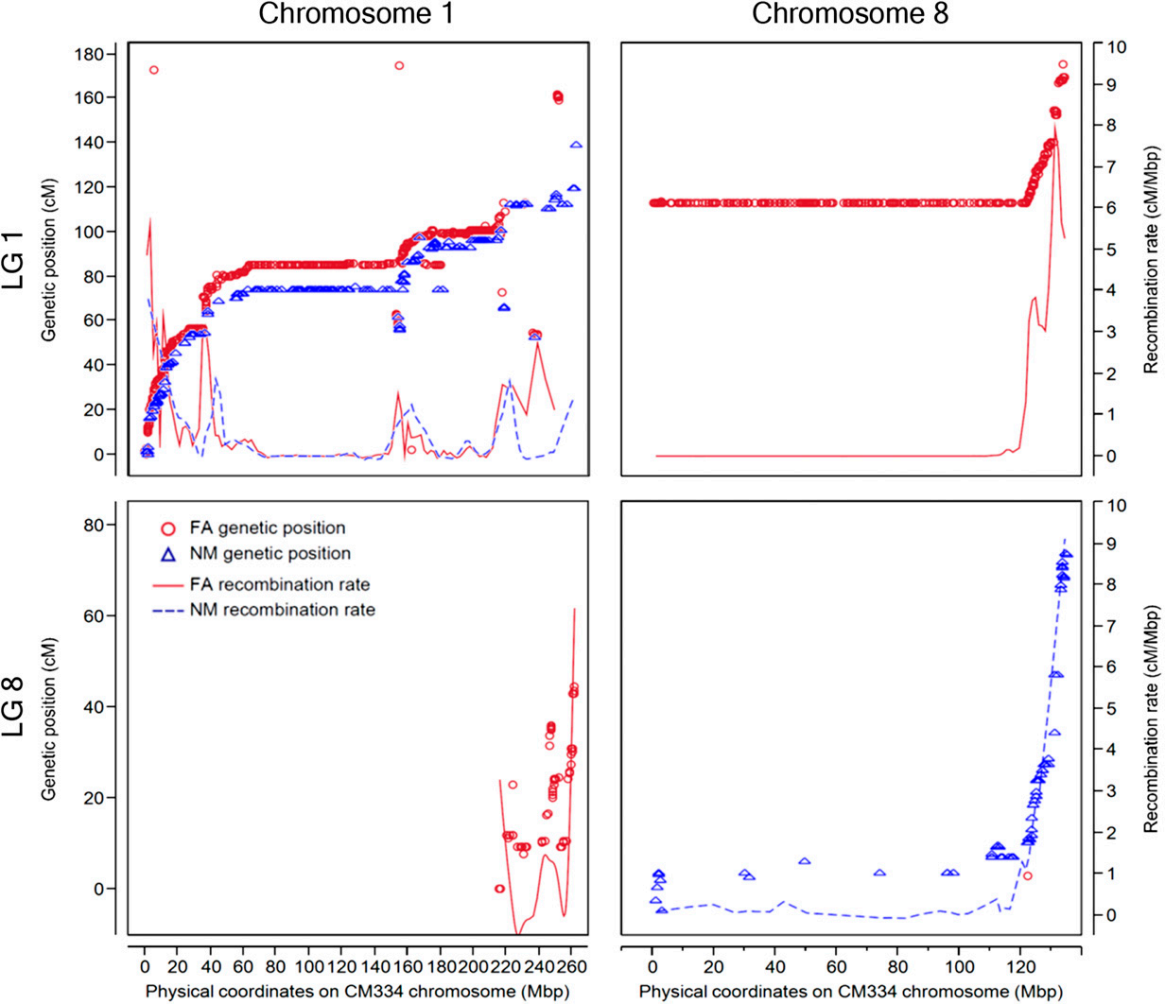
Relationship between physical and genetic positions with corresponding recombination rates for chromosomes 1 and 8. The 2240 FA and 387 NM map markers that have been placed on CM334 chromosomes 1 and 8. The left Y-axis indicates the linkage group (LG) and centimorgan position of FA (red circles) and NM (blue triangles) unigene markers. The X-axis represents the physical position (megabase pair) of unigenes on the pseudomolecules. The right Y-axis represents the recombination frequency in cM/Mbp along the chromosomes for the FA (red line) and NM (blue dash line) maps.

Of the 30,173 pepper unigenes represented on the Pepper GeneChip, 26,725 were identified at 98% identity or greater in the CM334 v1.5 genome assembly (Kim *et al.* 2014). A unique match for 25,472 unigenes (84%) was identified and 18,930 (62%) had been placed on chromosome pseudomolecules (Table S8 and File S4). There were 68% of the FA and 67% of the NM mapped unigenes placed on CM334 chromosomes with 98% (10,866) and 96% (2511) of these on shared linkage group/chromosome pairs, respectively (Table S9, Table S10, Figure 7, Figure 8, Figure S3, and Figure S4) (Kim *et al.* 2014). A query against the *C. annuum* ‘Zunla-1’ 2.0 assembly (Qin *et al.* 2014) found 23,848 EST unigenes that matched the Zunla genome sequences and positions for 23,332 unigenes were unambiguously identified with 21,194 (70%) placed on chromosome pseudomolecules (Table S11, Table S12; Figure S3, and Figure S4). There were 83% (13,488) and 81% (3,125) of the FA and NM markers identified in the assembly with 12,242 and 2773 found on chromosomes and 97% and 95% of these on common linkage group/chromosome pairs, respectively.

There were 3629 FA markers that matched CM334 sequences on chr00 (File S4). These sequences have been assembled into scaffolds. There were matches to 520 scaffolds, 390 scaffolds had multiple unigene matches, and 346 (89%) of those were to a single FA LG. A total of 330 scaffolds matched multiple unigene markers mapping within 5 cM and 301 scaffolds had markers mapping to a single genetic bin, accounting for 2507 and 779 FA markers, respectively.

The assignment of chromosomal positions was highly conserved between both maps and each genome, with collinearity coefficients of 0.999 and greater (Figure S4). However, six pseudomolecules of the Zunla assembly are inverted relative to the P3, 4, 6, 9, 11, and 12 linkage groups (Qin *et al.* 2014). The chromosomes were densely covered by the FA markers with only a few, small segments of physical positions not covered. On the basis of FA markers associated with the 3.02 Gb of assembled CM334 chromosomes, 1.39 Gb (46%) of the genome was nonrecombining. There were several physical segments devoid of NM markers, generally corresponding to large physical segments with little to no genetic recombination. In general, the two linkage maps displayed similar patterns of recombination with the distal regions of chromosomes showing greater recombination and the central, likely pericentromeric, regions with greatly reduced recombination. The acrocentric chromosomes 2 and 8 showed concomitant reduced recombination near the top of the chromosome.

### Syntenic analyses

**Figure 9:**
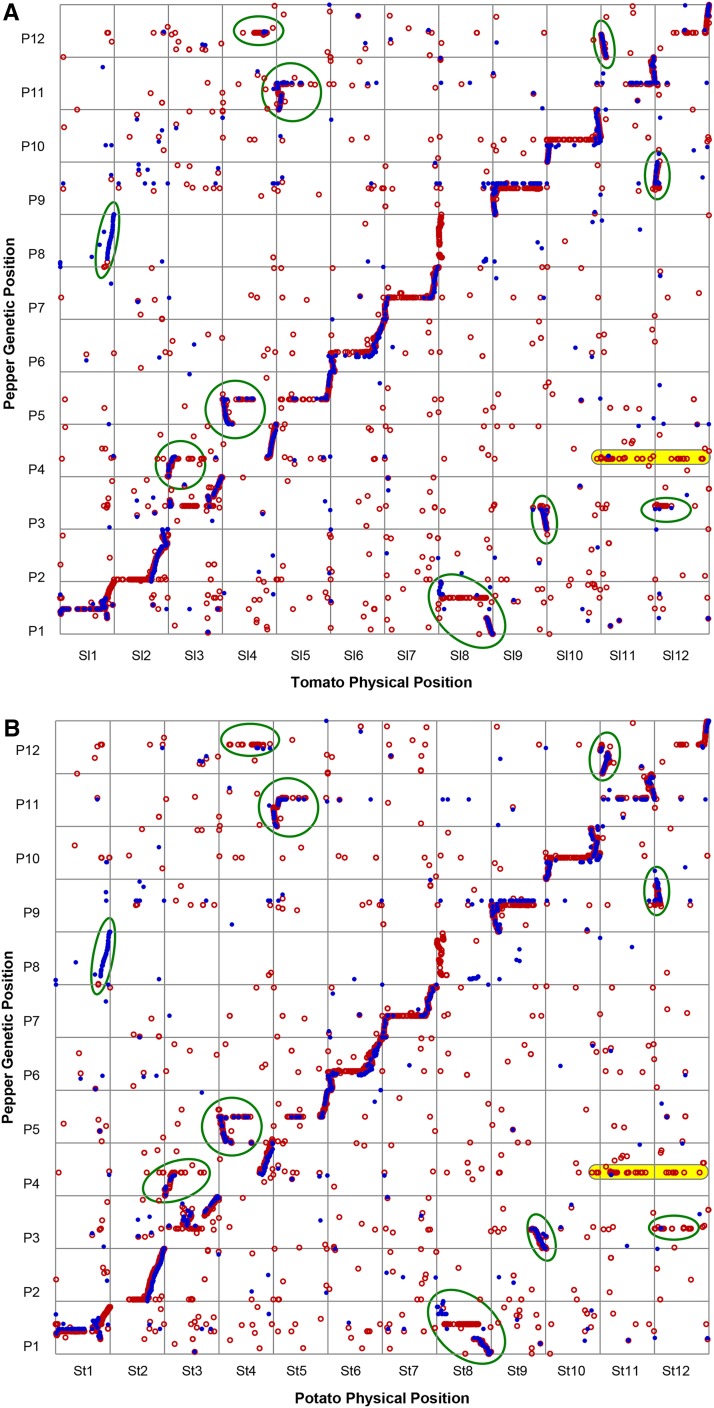
Pepper maps *vs.* tomato and potato genomes. Genetic and physical positions of FA (red circles) and NM (blue dots) markers matching tomato and potato genome sequences ≥ 80% ID and ≥75% unigene coverage. (A) Positions of 8267 mapped unigenes, 7660 FA and 1,755 NM found on tomato chromosomes. (B) Positions of 8131 mapped unigenes, 7551 FA and 1723 NM found on the potato chromosomes. Green circles represent previously identified translocations between pepper and tomato whereas the P4/Sl11 and St11 clusters (highlighted in yellow) represent a previously unmapped translocation event.

We analyzed synteny between each pepper map and the assembled genomes of pepper’s close solanaceous relatives, tomato and potato (File S5, Table S13, Table S14, Table S15, Table S16, and Figure 9). Alignment of the FA and NM maps to both genomes was similar, demonstrating good concordance between both pepper maps and the tomato and potato genomes. Dot plots showing relative pepper genetic positions *vs.* relative physical positions on each chromosome group are highly similar between the tomato and potato assemblies. These plots demonstrate several translocations in common between tomato and potato with pepper. In addition to the eight major translocations previously described (Tanksley *et al.* 1988; Livingstone *et al.* 1999; Wu *et al.* 2009; Wu and Tanksley 2010), there appears to be a translocation between the nonrecombining region on P4 and T11/S11 and possibly T12/S12 that has, to our knowledge, not been observed by syntenic analyses between pepper maps and tomato.

## Discussion

We constructed ultrahigh density EST-based genetic maps for *Capsicum* using GeneChip technology. An interrogation of 30,173 unigenes segregating in two RIL populations resulted in high-quality, comprehensive *Capsicum* genetic maps: a 16,780 unigene interspecific and a 3878 unigene intraspecific map with a total of 17,365 nonredundant unigenes mapped. The maps capture variation in fairly diverse germplasm, including a small fruited *C. frutescens* variety and *C. annuum* lines from three distinct gene pools, the semidomesticated disease-resistant CM334 variety and Jalapeño and Anaheim types. These maps enable genetic studies in *Capsicum* and comparisons between Solanaceous species. We have reported previously an immediate application of this resource for molecular breeding through high-resolution mapping of QTL for multiple traits (Brand *et al.* 2012; Yarnes *et al.* 2013; Naegele *et al.* 2013; Rehrig *et al.* 2014).

The haploid chromosome number for *Capsicum* is 12; however, when details have been reported for construction of pepper maps, *de novo* clustering of markers into 12 linkage groups has been rare, with upwards of 20 linkage groups being identified (Prince *et al.* 1993; Livingstone *et al.* 1999; Chaim *et al.* 2001; Lefebvre *et al.* 2002; Sugita *et al.* 2005; Barchi *et al.* 2007; Truong *et al.* 2010). Common pepper map markers, integrated maps, and syntenic relationships with tomato have been used to assemble multiple small groups into chromosomal linkage groups (Paran *et al.* 2004; Barchi *et al.* 2007; Mimura *et al.* 2012). As expected, the increase in marker density and coverage attributable to *de novo* identification of gene-based markers and the fairly even distribution of genes along pepper chromosomes (Kim *et al.* 2014) resulted in FA markers clustering into 12 linkage groups.

Initially, FA linkage groups P1 and P8 formed a single cluster due to pseudolinkage of markers on the translocated chromosomal arms between *C. annuum* and *C. frutescens*. This has been observed in previous interspecific maps between *C. annuum* and *C. frutescens* or *C. chinense* (Livingstone *et al.* 1999; Wu *et al.* 2009; Park *et al.* 2014). Our use of CheckMatrix to visualize recombination patterns provided an obvious demarcation between the P1 and P8 linkage group, although pseudolinkage prohibited the chromosomal assignment of a few markers near the translocation break point without the guidance of the intra-specific NM map that lacks the translocations. A comparison of both maps indicates the P1/P8 translocation break point is coincident with the most marker dense genetic bin, in an approximately 120 Mb nonrecombining region, of P1. Identifying the precise physical position of the breakpoint may be difficult without whole genome sequencing of the RILs.

The NM map data, having less than one fourth the markers and about half of the population size of the FA, was insufficient for clustering markers into 12 groups. This also may explain the inability to map 30% of the NM polymorphic unigenes, a phenomenon common to previous pepper mapping efforts, particularly for intraspecific maps of *C. annuum* (Prince *et al.* 1993; Livingstone *et al.* 1999; Kang *et al.* 2001; Barchi *et al.* 2007). The common map markers indicate apparent errors in marker grouping can occur when mapping members of multigene families. This would be more pronounced when detecting polymorphisms in more closely related lines.

Marker ordering was carried out *de novo* for each map independently and resulted in very high collinearity between maps. The lowest collinearity coefficient was between P8 wild and P1 cultivated, consistent with pseudolinkage (Livingstone *et al.* 2000). In addition, there were some isolated differences in marker ordering, which tended to be at the top or bottom of linkage groups. Marker ordering can be influenced by missing calls or calling errors, particularly in high-density maps (Hackett and Broadfoot 2003). The SPP detection algorithms cannot discriminate heterozygous alleles from homozygotes, which likely resulted in both missing and erroneous calls (Hill *et al.* 2013). Because individual calls were collapsed into consensus haplotypes for each unigene and then consensus haplotypes for each genetic bin before calculating distance, heterozygous alleles could be determined by their high percentage of missing values and low A or B allele frequencies. Overall, missing or erroneous calls seem to have had little impact on original marker ordering, which had little change during subsequent mapping iterations where heterozygous call corrections were implemented.

Marker distribution was similar between chromosomes for both maps; however, marker distribution on P7 had unique features in each map. The NM linkage group 7 had the largest marker interval, lowest marker density and, compared with the CM334 chromosome 7, had the smallest fraction of unigene markers per gene (Kim *et al.* 2014). These features all indicate reduced polymorphism on P7 between the intraspecific NM parents. Reduced polymorphism was not apparent on FA P7. However, this was the only linkage group with multiple 1-cM bins having more than 300 markers. The two marker-dense bins were separated by more than 50 cM and corresponded to both the pericentromeric region and region of reduced recombination near the distal end of chromosome 7. Similarly, the COSII map (F2 of the FA RIL population) had a large gap (>20 cM) on P7 separating two clusters of markers. The reduced recombination indicated the possibility of a paracentric inversion between the COSII parents (Wu *et al.* 2009). However, marker collinearity between the FA and NM markers on P7 did not support a paracentric inversion between FA parents. Alternatively, the high density of markers near the distal end of the chromosome points to increased variation, resulting in reduced recombination near the subtelomeric region of chromosome 7 between *C. frutescens* and *C. annuum*. Chromosome subtelomeric regions can have high rates of sequence variation and have been shown to be important for recombination (Abecasis *et al.* 2010; Calderon *et al.* 2014). Whether this is related to the reduced polymorphism in the intraspecific population is unclear.

The total lengths of the FA and NM maps were similar, close to the 1390-cM map length estimated by Lefebvre *et al.* (1995). The FA map was 19 cM (1.4%) shorter than the NM map, consistent with the expectation of reduced recombination in the interspecific population. However, there were five linkage groups that were longer in the FA map. This finding could be explained by incomplete chromosomal coverage at the ends as the result of reduced polymorphism, expansion caused by segregation distortion, or elevated heterozygosity. Differences of chromosomal coverage could be determined by comparing the relative positions of markers common to both maps at the top and bottom of linkage groups. Of the five FA linkage groups that were longer than their NM counterparts, FA markers on P2, P5, and P9 extended further by a total of 30 cM, which would result in a relative underestimation of NM length. Significant segregation distortion was observed in 10 FA linkage groups, including severe marker distortion on P2 favoring the *C. frutescens* allele at the top and the NuMex RNaky allele at the bottom which may result in the overestimation of recombination. Also, contrary to expectation, comparisons between the two maps and the maps with the genome indicate that the bottom of chromosome 4 and tops of chromosomes 3, and 6 appear to have less recombination in the NM *vs.* FA map. Overall, these features suggest that both chromosomal coverage at the ends and segregation distortion may have contributed in part to the underestimation of NM length and/or overestimation of FA length for several linkage groups. However, the linkage group with the largest expansion in the FA map was P3 and this appears to be primarily due to reduced recombination at the top of NM P3.

Comparisons between genetic distances and physical distances showed similar patterns of recombination rate variation along the pepper chromosomes. Strong recombination repression occurs over long chromosomal regions, which likely correspond to pericentromeric regions. This pattern of recombination also has been observed in tomato and other species (Ganal *et al.* 2011; Huo *et al.* 2011; Sim *et al.* 2012; Zhang *et al.* 2012; Deokar *et al.* 2014). However, recombination repression appears to be more pronounced in pepper, most similar to what has been observed in tomato, suggesting that it may be a common feature in the Solanaceae. Despite fairly even genetic map coverage, there were several large gaps in chromosomal coverage by NM markers on all linkage groups except P1, P9, and P11. These gaps corresponded to the nonrecombining regions, also demonstrated by the absence of the extremely marker dense 1 cM bins that were observed in FA linkage groups. This shows that although there are polymorphic genes present in these chromosomal regions in the FA population, there was a smaller proportion of NM polymorphic genes in the nonrecombining regions. Regions having low recombination have been shown to have less variation in both plants and animals (Begun and Aquadro 1992; Buckler and Thornsberry 2002; Lercher and Hurst 2002; Nachman 2002; Roselius *et al.* 2005). This, along with the fourfold reduction in NM *vs.* FA markers explains, at least in part, the gaps in chromosomal coverage by NM markers. Confounding this would be the potential for gaps in the genome assembly across these regions, because scaffold ordering cannot benefit from genetic maps.

Syntenic analyses between the FA and NM maps and the tomato (Sl) and potato (St) genome assemblies confirm 10 translocations that were previously reported (Livingstone *et al.* 1999; Wu *et al.* 2009; Wu and Tanksley 2010; Qin *et al.* 2014). Additional translocations were observed between P4 and SlChr11/StChr11 and P4 and SlChr12/StChr12. Both of these translocations correspond to the nonrecombining region on P4 and were only observed with the greater density FA map and the genome assembly of Qin *et al.* (2014) in a detailed comparison of the pepper and tomato genomes. Because the intraspecific maps used to assemble the pepper genomes had half the markers of the full FA map, and we observed reduced polymorphism in the nonrecombining regions of the intraspecific NM map, the P4 centromeric region may not be completely assembled at this time. Interestingly, there is also a translocation of the top arm of P4, including markers from the nonrecombining region that correspond to the top of SlChr03/StChr03. This finding indicates that there have been as many as three translocation events between pepper and tomato/potato within the nonrecombining region of P4, requiring five chromosomal breakages.

Most pepper maps to-date are primarily based on anonymous markers and thus difficult to compare in many cases. Because the maps presented here are gene-based, they can be easily associated with other gene-based genetic maps regardless of the polymorphic positon(s) within the gene. Thus, trait-marker associations and candidate gene identification will be facilitated and easily assayed across populations. The utility of the maps has already been demonstrated by their use in an in-depth study of 39 phenological, leaf, fruit, morphological, and capsaicinoid traits and identification of a *Phytophthora* resistance gene candidate (Brand *et al.* 2012; Yarnes *et al.* 2013; Naegele *et al.* 2013; Rehrig *et al.* 2014). In addition, the maps were used to aid assembly of the recently released *Capsicum* reference genome based on CM334 (Kim *et al.* 2014). Even so, we found more than 300 unassembled scaffolds that were each assigned to a single genetic bin by FA map markers. This gives approximate chromosomal positions for these scaffolds and their gene compliments, providing critical information for candidate gene identification. These maps will enable further trait associations and gene cloning in *Capsicum* as well as comparisons with gene-based genetic maps and genome assemblies across species.

## 

## Supplementary Material

Supporting Information
