# Supplementary material for: Ultra-High Density, Transcript-Based Genetic Maps of Pepper Define Recombination in the Genome and Synteny Among Related Species
Source: G3 (Bethesda). 2015 Sep 8;5(11):2341–55. doi: 10.1534/g3.115.020040 (PMC4632054; doi:10.1534/g3.115.020040)
Supplement: Supporting Information [file supp_5_11_2341__index.html]

Ultra-High Density, Transcript-Based Genetic Maps of Pepper Define Recombination in the Genome and Synteny Among Related Species — Supporting Information 

# Ultra-High Density, Transcript-Based Genetic Maps of Pepper Define Recombination in the Genome and Synteny Among Related Species

## Supporting Information for Hill *et al.*, 2015

**Files in this Data Supplement:**

- Supporting Information - Tables S1-S16, Figures S1-S4, and descriptions of Files S1-S5 (PDF, 1 MB)
- Table S1 - Summary of overall allele counts. (PDF, 124 KB)
- Table S2 - Distribution of genetic bin types. (PDF, 123 KB)
- Table S3 - Size and positions of largest 1 cM bins. (PDF, 124 KB)
- Table S4 - NM regions with segregation distortion. (PDF, 127 KB)
- Table S5 - FA regions with segregation distortion. (PDF, 127 KB)
- Table S6 - QTL overlapping with regions of skewness in the FA population. (PDF, 124 KB)
- Table S7 - The number of common markers for each NM and FA linkage group pair. (PDF, 128 KB)
- Table S8 - Summary of GMAP results for Chip assembly (unigenes) versus CM334 v1.5 and Zunla-1 v2.0 genome assemblies. (PDF, 129 KB)
- Table S9 - NM map vs CM334 v1.5 genome. (PDF, 129 KB)
- Table S10 - NM map vs Zunla-1 v2.0 genome. (PDF, 129 KB)
- Table S11 - FA map vs CM334 v1.5 genome. (PDF, 129 KB)
- Table S12 - FA map vs Zunla-1 v2.0 genome. (PDF, 101 KB)
- Table S13 - NM map vs Tomato v2.5 genome. (PDF, 128 KB)
- Table S14 - NM map vs Potato v 2.06 genome. (PDF, 128 KB)
- Table S15 - FA map vs Tomato v2.5 genome. (PDF, 128 KB)
- Table S16 - FA map vs Potato v 2.06 genome. (PDF, 128 KB)
- Figure S1 - Distance between crossovers for the NM (A) and FA (B) genetic maps. (PDF, 640 KB)
- Figure S2 - Comparative maps between *C. frutescens* acc. BG2814-6 × *C. annuum* 'NuMex RNaky' (FA) and *C. annuum* 'Early Jalapeño' × *C. annuum* 'CM344' (NM) RIL populations. (PDF, 781 KB)
- Figure S3 - Pepper maps vs. pepper genome assemblies. (PDF, 452 KB)
- Figure S4 - Regression of marker order between mapped unigenes on common linkage group/chromosome pairs. (PDF, 577 KB)
- File S1 - Data tables for *C. frutescens* acc. BG2814-6 × *C. annuum* 'NuMex RNaky' (FA07) map. (.xlsb, 3 MB)
- File S2 - Data tables for *C. annuum* 'Early Jalapeño' × *C. annuum* 'CM344' (NM06) map. (.xlsb, 883 KB)
- File S3 - Common map markers. (.xlsb, 118 KB)
- File S4 - Maps vs. pepper assemblies. (.xlsb, 2 MB)
- File S5 - Maps vs. tomato and potato assemblies. (.xlsb, 981 KB)
