# Supplementary material for: Ultra-High Density, Transcript-Based Genetic Maps of Pepper Define Recombination in the Genome and Synteny Among Related Species
Source: G3 (Bethesda). 2015 Sep 8;5(11):2341–55. doi: 10.1534/g3.115.020040 (PMC4632054; doi:10.1534/g3.115.020040)
Supplement: Supporting Information [file supp_g3.115.020040_020040SI.pdf]

G3 · Genes | Genomes | Genetics

Ultra-high density, transcript-based genetic maps of pepper define recombination in the genome and synteny among related species

Theresa Hill*§, Hamid Ashrafi*§, Sebastian Reyes Chin-Wo†, Kevin Stoffel*, Maria-Jose Truco†, Alexander Kozik†, Richard Michelmore†§, and Allen Van Deynze*§[1]

*Seed Biotechnology Center, University of California, Davis, California 95616
†The Genome Center, University of California, Davis, California 95616
§Department of Plant Sciences, University of California, Davis, California 95616

[1]Corresponding author: Seed Biotechnology Center, University of California, Davis, California 95616. E-mail: avandeynze@ucdavis.edu

DOI: 10.1534/g3.115.020040

Table S1. Summary of overall allele counts.

	# Calls	A	B	H	Missing
NM Bins	49,329	24,867 (50.4)	23,335 (47.3)	781 (1.6)	346 (0.7)
FA Bins	250,852	123,708 (49.3)	114,560 (45.7)	11,032 (4.4)	1,552 (0.6)

Table S2. Distribution of genetic bin types

	NM map<br>Genetic Bins	FA map<br>Genetic Bins
Singltons	290	779
Multiple markers	493	1326
Total	783	2105

Table S3. Size and positions of largest 1 cM bins.

	FA map			NM Map		
Linkage Group	Maximum 1 cM Bin Size	Position (cM)	% Markers	Maximum 1 cM Bin Size	Position (cM)	% Markers
P1	624	138	20%	82	84	18%
P2	286	5	17%	46	52	11%
P3	271	65	13%	34	75	8%
P4	279	46	23%	24	87	10%
P5	220	47	23%	55	46	22%
P6	214	52	15%	29	38	10%
P7	375	45	31%	25	48	17%
P8	21	13	9%	28	12	11%
P9	250	58	25%	282	60	54%
P10	238	44	22%	27	42	9%
P11	243	45	24%	177	51	49%
P12	276	45	25%	25	120	11%

Table S4. NM regions with segregation distortion.

LG	Direction	Range (cM)	Span (cM)
1	Early Jalapeño	120-120	< 1
3	CM334	58-64	6
4	Early Jalapeño	0-5	5
5	CM334	15-23	8
	Early Jalapeño	87-91	4
6	CM334	13-32	19
	Early Jalapeño	95-104	9
7	Early Jalapeño	38-38	< 1
	Early Jalapeño	100-111	11
9	CM334	2-2	< 1
	Early Jalapeño	47-63	16
	Early Jalapeño	90-95	5
10	Early Jalapeño	54-55	1
	Early Jalapeño	128-128	< 1
11	Early Jalapeño	0-14	14

Table S5. FA regions with segregation distortion.

LG	Direction	Range (cM)	Span (cM)
1	*C. frutescens*	0-2	2
	NuMex RNaky	22-70	48
	NuMex RNaky	104-112	8
2	*C. frutescens*	0-22*	22
	NuMex RNaky	56-59	3
	NuMex RNaky	67-72*	5
	NuMex RNaky	76-95*	19
4	NuMex RNaky	78-94*	16
	NuMex RNaky	100-131*	32
5	NuMex RNaky	20-21	1
	NuMex RNaky	65-70	5
6	NuMex RNaky	41-46*	5
	NuMex RNaky	78-83	4
	NuMex RNaky	91-95	4
7	NuMex RNaky	10-11	1
	NuMex RNaky	21-22	1
	C. frutescens	47-52	5
	NuMex RNaky	105-107	2
9	NuMex RNaky	45-78	33
	NuMex RNaky	84-107*	23
10	NuMex RNaky	40-40	<1
	NuMex RNaky	53-70	17
11	*C. frutescens*	18-18	<1
	C. frutescens	29-49	19
12	*C. frutescens*	32-58	25

*Regions that include FA QTL listed in Table S6.

Table S6. QTL overlapping with regions of skewness in the FA population.

Direction of Skewness	FA QTL Trait	LG	Peak (cM)	LOD	R2	P value	Additive
C. frutescens	Branching Density	2	0.7	3.9	0.120	0.025	-0.31
NuMex Rnaky	Days to Breaker	2	70.4	5.1	0.174	0.005	-6.32
NuMex Rnaky	Days to Breaker	2	80.9	8.3	0.269	0.001	-8.16
NuMex Rnaky	Branching Density	4	81.8	6.4	0.182	0.001	0.28
NuMex Rnaky	Stigma Exsertion	4	113.1	3.6	0.112	0.030	-0.13
NuMex Rnaky	Days to Flowering	6	39.8	5.1	0.153	0.005	-3.84
NuMex Rnaky	Days to Flowering	9	97.5	5.9	0.180	0.001	-4.32

Tables S7. The number of common markers for each NM and FA linkage group pair.

	NM LGs												
FA LGs	1	2	3	4	5	6	7	8	9	10	11	12	Total
1	248		1	2	1	2	1	177	2	1	2		437
2	1	265	1		1	1	1		1	1		1	273
3	2	1	274	1			1	2			2	1	284
4	1	2		173		1	1	1	1		2		182
5	2		1	1	166	1		2	1				174
6		1				199			2		1		203
7		3	1				91		1	1		1	98
8	56				1			1	1				59
9		2	1		1		1	1	364				370
10		1	1		2				1	196			201
11	1		2					1			234		238
12	2				1				2	1	2	140	148
Total	313	275	282	177	173	204	96	185	376	200	243	143	2,667
Same LG	97%	96%	97%	98%	96%	98%	95%	96%	97%	98%	96%	98%	97%

Table S8. Summary of GMAP results for Chip assembly (unigenes) versus CM334 v1.5 and Zunla-1 v2.0 genome assemblies. The number of unigenes mapped to any linkage group identified on each chromosome pseudomolecule is also shown.

	CM334 v1.5			Zunla-1 v2.0		
Target	ESTs	NM markers	FA markers	ESTs	NM markers	FA markers
Chr01	2258	292	1334	2477	311	1429
Chr02	2348	321	1382	2347	331	1384
Chr03	2334	296	1379	2800	321	1632
Chr04	1182	116	714	1594	166	930
Chr05	1175	177	680	1333	193	762
Chr06	1498	180	843	1911	184	1078
Chr07	1622	101	947	1363	102	787
Chr08	1115	108	725	1881	195	1171
Chr09	1169	365	670	1314	361	733
Chr10	1407	222	815	1398	226	777
Chr11	1207	264	654	1242	213	667
Chr12	1615	176	926	1534	170	892
Chr 1-12	18930	2618	11069	21194	2773	12242
Chr00	6542	820	3629	2138	352	1246
Total	25472	3438	14698	23332	3125	13488

Table S9. NM map vs CM334 v1.5 genome. The number of map markers placed on CM334 pseudomolecules for each linkage group/chromosome pair. Unigenes on the same linkage group as chromosome were used to calculate the coefficients of colinearity and recombination rates.

**CM334**	**NM Linkage Group**												
**Chr**	**1**	**2**	**3**	**4**	**5**	**6**	**7**	**8**	**9**	**10**	**11**	**12**	**Total**
1	273		3	2				11	1	1		1	292
2	2	314	1	1				1				2	321
3	3		286	1	2				3		1		296
4	2			112			2						116
5	3				164	9		1					177
6			1			176				1	2		180
7		2					95	1	1	1		1	101
8	3		1			1		99	2		1	1	108
9	1	1						1	362				365
10									3	207	12		222
11	1		1					4			258		264
12	1		1		1			2	2	1	3	165	176
Assembled	289	317	294	116	167	186	97	120	374	211	277	170	2618
Chr00	102	52	75	100	56	60	33	114	89	61	47	31	820
Total	391	369	369	216	223	246	130	234	463	272	324	201	3438
% Chr/LG Match	0.94	0.99	0.97	0.97	0.98	0.95	0.98	0.83	0.97	0.98	0.93	0.97	0.96

Table S10. NM map vs Zunla-1 v2.0 genome. The number of map markers placed on Zunla pseudomolecules for each linkage group/chromosome pair. Unigenes on the same linkage group as chromosome were used to calculate the coefficients of colinearity.

Zulna-1	NM Linkage Group												
**Chr**	**1**	**2**	**3**	**4**	**5**	**6**	**7**	**8**	**9**	**10**	**11**	**12**	**Total**
1	293					3		7	7		1		311
2	1	326	1					1	1			1	331
3	2	1	309	1	4			1	1		2		321
4	2	1	1	158			1		1		2		166
5	3			2	171	1		2			6	8	193
6	1			1		179		1			2		184
7		1					96		3	1	1		102
8	2					1	1	187	1	2	1		195
9							1	1	359				361
10				10	5				1	210			226
11	1		1					1	9		201		213
12	1					2		6			3	158	170
Assembled	306	329	312	172	180	186	99	207	383	213	219	167	2773
Chr00	51	15	23	30	18	39	17	8	49	35	56	11	352
Total	357	344	335	202	198	225	116	215	432	248	275	178	3125
% Chr/LG Match	0.96	0.99	0.99	0.92	0.95	0.96	0.97	0.90	0.94	0.99	0.92	0.95	0.95

Table S11. FA map vs CM334 v1.5 genome. The number of map markers placed on CM334 pseudomolecules for each linkage group/chromosome pair. Unigenes on the same linkage group as chromosome were used to calculate the coefficients of colinearity and recombination rates.

CM334	FA linkage group												
**Chr**	**1**	**2**	**3**	**4**	**5**	**6**	**7**	**8**	**9**	**10**	**11**	**12**	**Total**
1	1182	1	4	6			8	125		3	1	4	1331
2	1	1363	2	1	3	5	3			1	2	1	1383
3	2		1365	3	4	2			2			1	1381
4		1	1	710						1		1	717
5				1	660	15	1			2	1		685
6	2	3	5	2		827	1		1	1	1		849
7	4	1	5	1		1	928			3	1	3	951
8	716		3		1	1		1	1			2	731
9	3	2		2			2		659		1	1	678
10	1	1	1				5		2	787	18		825
11	15	2	2			2	1		1		631		665
12	1	1	1	1		3	2		2	2	1	912	26
Assembled	1927	1375	1389	727	668	856	951	126	668	800	657	925	11069
Chr00	954	184	458	386	208	481	153	91	200	176	231	107	3629
Total	2881	1559	1847	1113	876	1337	1104	217	868	976	888	1032	14698
% Chr/LG Match	0.98	0.99	0.98	0.98	0.99	0.97	0.98	1.00	0.99	0.98	0.96	0.99	0.98

Table S12. FA map vs Zunla-1 v2.0 genome. The number of map markers placed on Zunla-1 pseudomolecules for each linkage group/chromosome pair. Unigenes on the same linkage group as chromosome were used to calculate the coefficients of colinearity.

Zunla-1	FA linkage group												
Chr	1	2	3	4	5	6	7	8	9	10	11	12	Total
1	1256	1		1		9	3	141	6	5	6	1	1429
2	1	1366	9	1		1	1		1	2	1	1	1384
3	5	6	1585		13	1	4		4	12	1	1	1632
4	11	1	1	910		1	2	1			2	1	930
5	14		3	2	687	1	5			2	20	28	762
6	13	1	6	1	1	1046	1			1		8	1078
7	2	2	2		6	1	752		10	4	5	3	787
8	1161	1	3			3		1		1		1	1171
9	10		1			1	2		718			1	733
10	3		13	11	4		2		2	742			777
11	2	1		3		1	1		11	2	646		667
12	5		3		4	3	1		1	3		872	892
Assembled	2483	1379	1626	929	715	1068	774	143	753	774	681	917	12242
Chr00	154	70	102	112	87	176	194	35	67	103	110	36	1246
Total	2637	1449	1728	1041	802	1244	968	178	820	877	791	953	13488
% Chr/LG Match	0.97	0.99	0.97	0.98	0.96	0.98	0.97	0.99	0.95	0.96	0.95	0.95	0.97

Table S13. NM map vs Tomato v2.5 genome. The number of map markers placed on tomato pseudomolecules (Sl 2.5) for each linkage group/chromosome pair.

Tomato	NM Linkage Group												
Chr	1	2	3	4	5	6	7	8	9	10	11	12	Total
1	103		1	1	1	1	1	117	2	2	1		230
2	2	196	4			1	2	1	7	3		1	217
3	4		105	42	2	1	1		3	1		2	161
4	3	2	1	57	57				2	3	2	9	136
5		1	2	7	46	1			2	1	57	1	118
6	1	2			1	106	1	2	1	1	2	3	120
7		2	2	2		2	56		2			2	68
8	70	1						23	4		3		101
9	1	3	75				2	3	139		3	1	227
10	2	1	1		2	2		1	3	114	1		127
11	2			9	1			2	1	1	80	37	133
12	4	1	4	1	3		5		49	5	3	37	112
Total	192	209	195	119	113	114	68	149	215	131	152	93	1,750

Table S14. NM map vs Potato v 2.06 genome. The number of map markers placed on potato pseudomolecules for each linkage group/chromosome pair.

Potato	NM Linkage Group												
Chr	1	2	3	4	5	6	7	8	9	10	11	12	Total
1	103	1	2	1	1	3	1	111	3	2	1		229
2	2	217	4			2	1	1	6	1		1	235
3	4	1	101	38	1		1		1			3	150
4	2	2		60	53				3		2	9	131
5		1	1	8	45				2		55	1	113
6	1	2			1	99	3	2	1		2	2	113
7	2	3	3	2		2	56		2			1	71
8	74							27	4		3		108
9	2	3	64		1			4	132		2	1	209
10	3		3			2			3	119		1	131
11	1		1	9		1		2	1	1	73	37	126
12	3	1	3	1	2	1			52	2	2	36	103
Total	197	231	182	119	104	110	62	147	210	125	140	92	1,719

Table S15. FA map vs Tomato v2.5 genome. The number of map markers placed on tomato (Sl 2.5) pseudomolecules for each linkage group/chromosome pair.

Tomato	FA Linkage Group												
Chr	1	2	3	4	5	6	7	8	9	10	11	12	Total
1	1,051	4	7	6	2	2	1	5	2	2		4	1,086
2	5	820	6	4		2	7		3	4	3	3	857
3	15	5	684	95	1	4			8	3	1	9	825
4	9	2	2	384	196	3	4	2	2	7	7	82	700
5	5	6	1	11	184	3	4		3	1	233	8	459
6	16	4	5	4	1	612	4	1	5	2	5	3	662
7	9	14	7	4	4	4	551		8	2	1	7	611
8	437	4	2	6	2	4	3	78	3	2	4	3	548
9	8	5	239	4	3	4	3		260	6	2	3	537
10	12	5	4	2	4	2	3	1	3	434	1	1	472
11	5	4	3	69	5	4	1		3	5	191	173	463
12	4	3	57	20	3		3	1	129	6	5	201	432
Total	1576	876	1017	609	405	644	584	88	429	474	453	497	7,652

Table S16. FA map vs Potato v 2.06 genome. The number of map markers placed on potato pseudomolecules for each linkage group/chromosome pair.

Potato	FA Linkage Group												
Chr	1	2	3	4	5	6	7	8	9	10	11	12	Total
1	1,071	4	6	3	4	3	1	2		6	1	6	1,107
2	6	838	6	7	1	2	6		2	3	2	3	876
3	15	10	657	77	3	4			6	2	2	4	780
4	8	3	2	372	181	3	3	3	1	4	5	82	667
5	5	6	2	12	184	4	5		5	2	225	7	457
6	20	3	5	3	1	606	5		4	4	1	2	654
7	10	10	8	4	3	4	534		5	3	2	9	592
8	433	2	1	8	1	4	3	78	3	3			536
9	13	6	231	6	3	4	3		244	3	1	3	517
10	8	3	5	5	1	2	3		3	427		1	458
11	5	3	4	67	3	8	3		2	4	178	165	442
12	2	4	75	17	4	2	2	1	132	3	5	210	457
Total	1,596	892	1,002	581	389	646	568	84	407	464	422	492	7,543

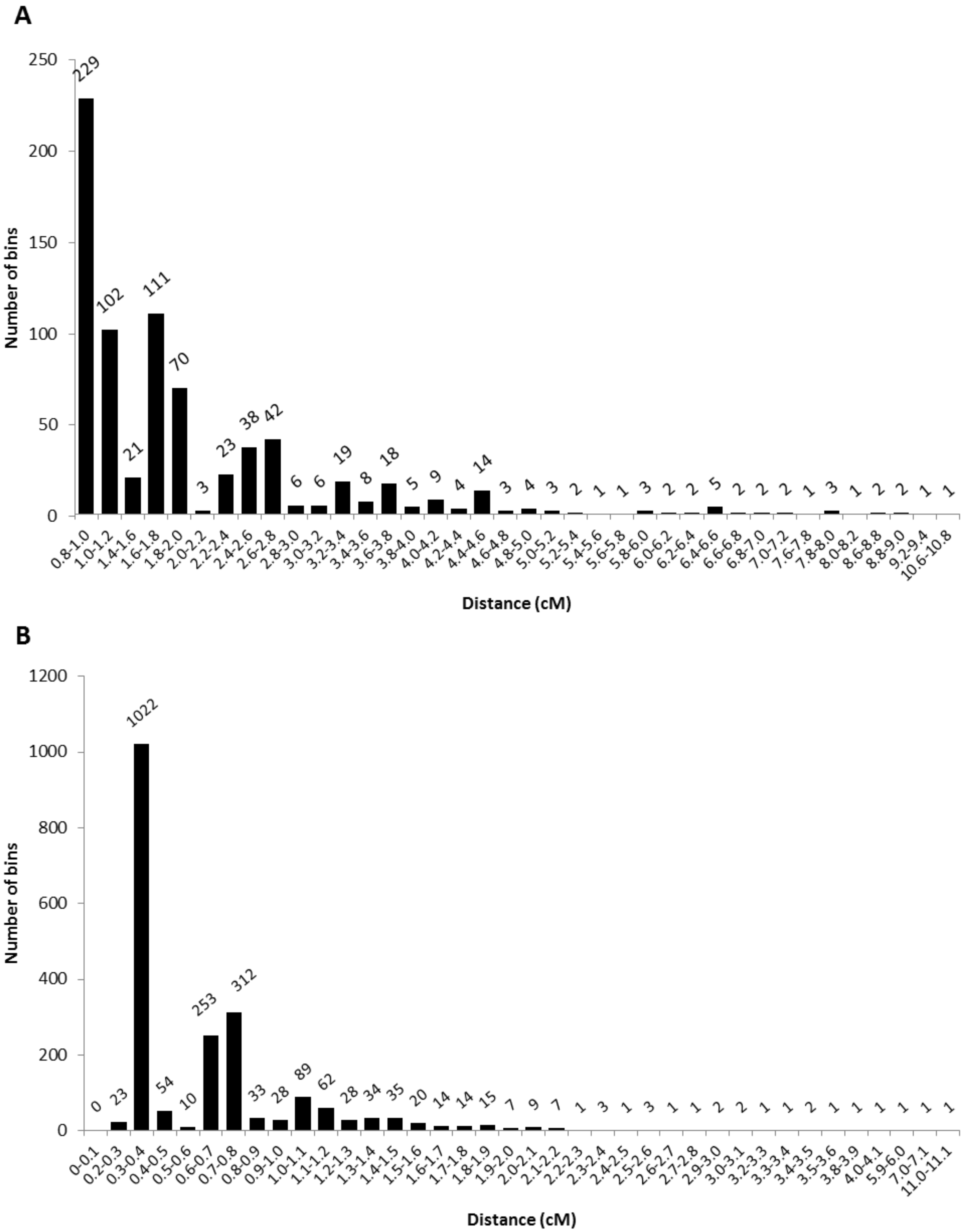

Figure S1. Distance between crossovers for the NM (A) and FA (B) genetic maps. Histograms representing the number of crossovers observed for each 0.25 cM increase in the gap between crossovers.

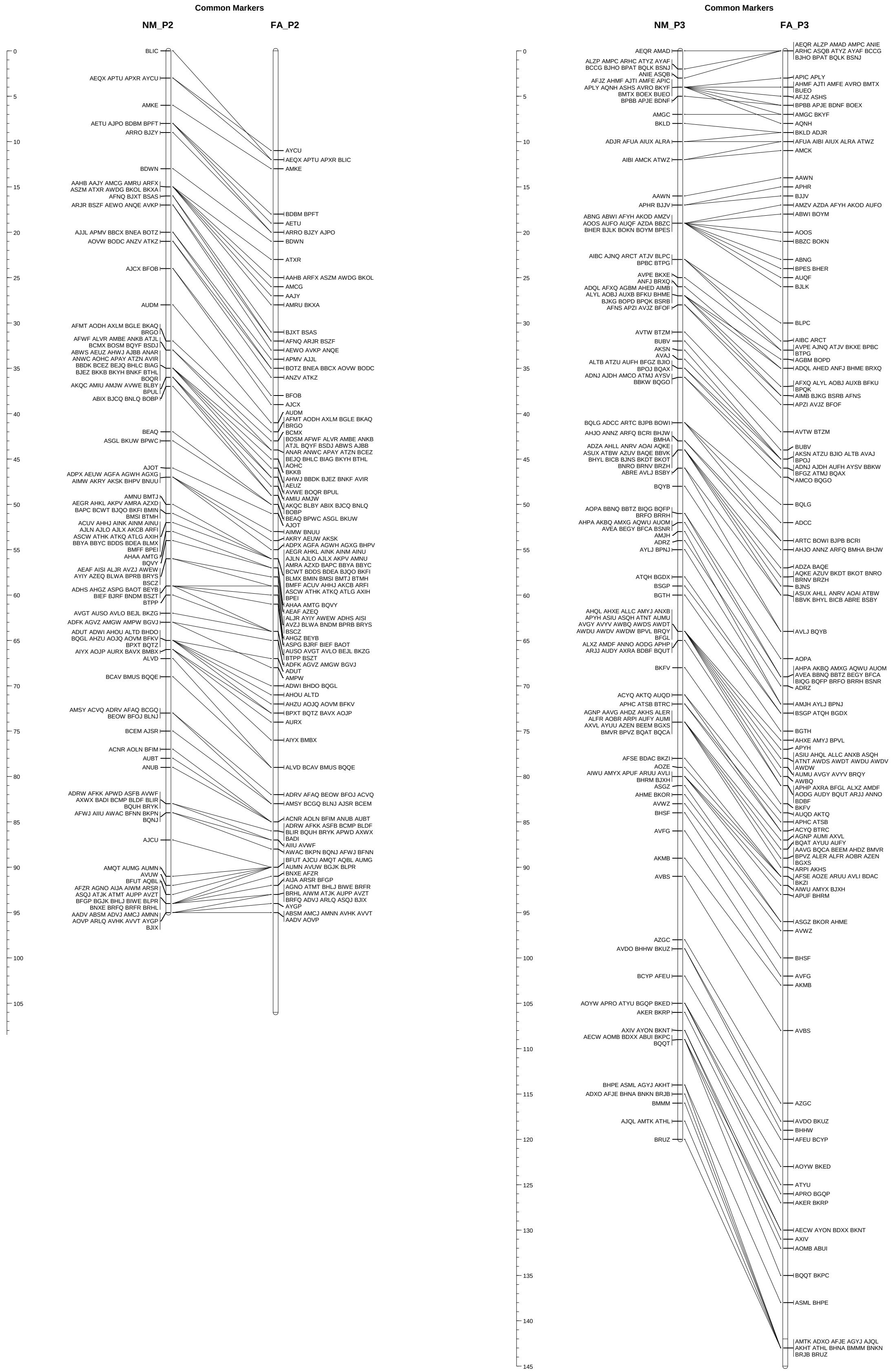
Common Markers
NM_P2
FA_P2
Common Markers
NM_P3
FA_P3

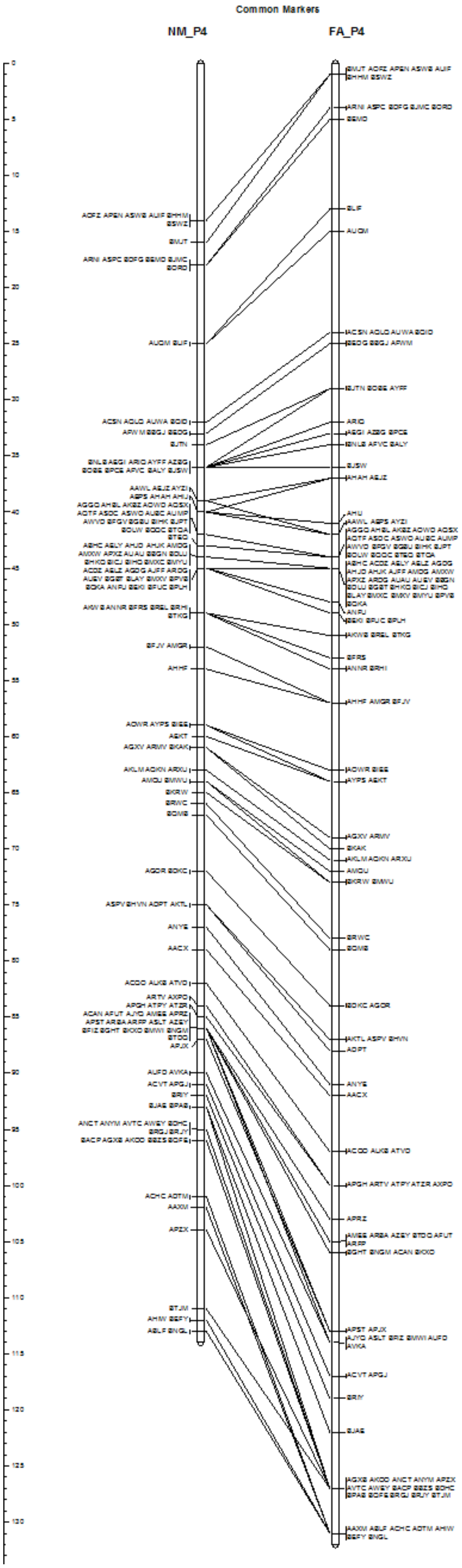
Common Markers
NM_P4
FA_P4

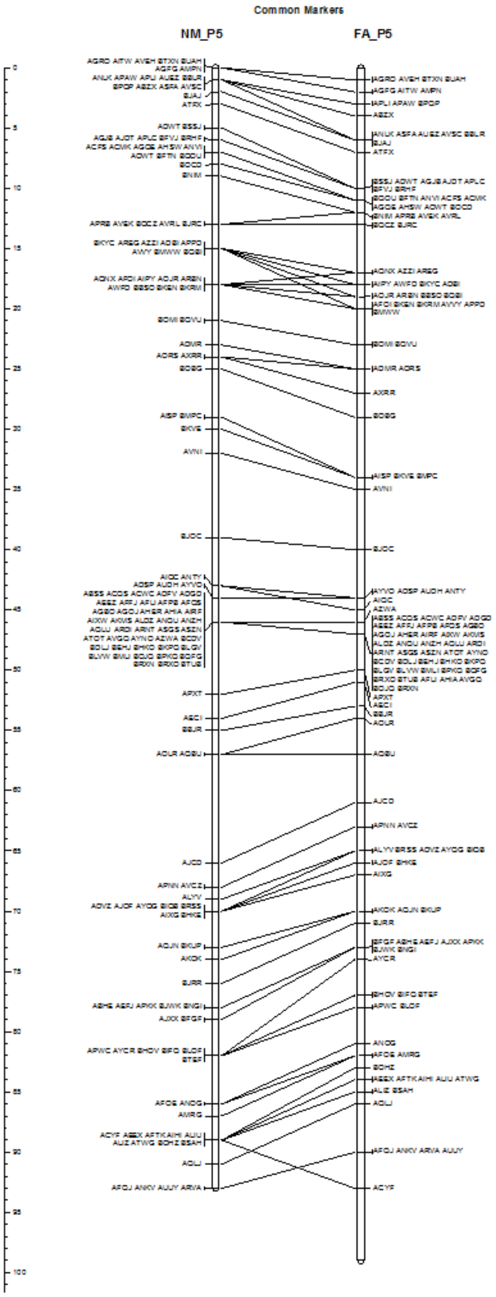
Common Markers
NM_P5
FA_P5

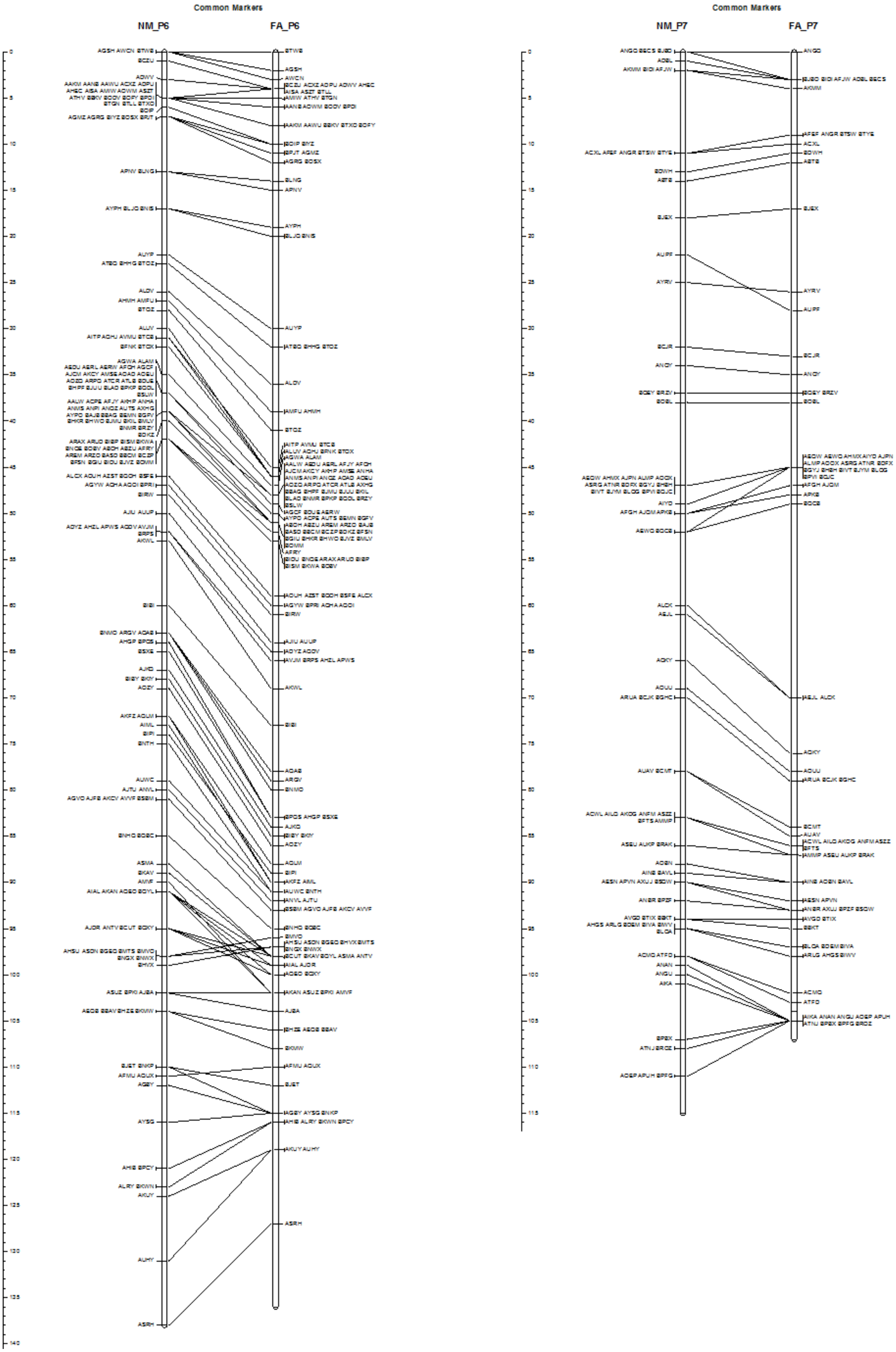
Common Markers
NM_P6
FA_P6
Common Markers
NM_P7
FA_P7

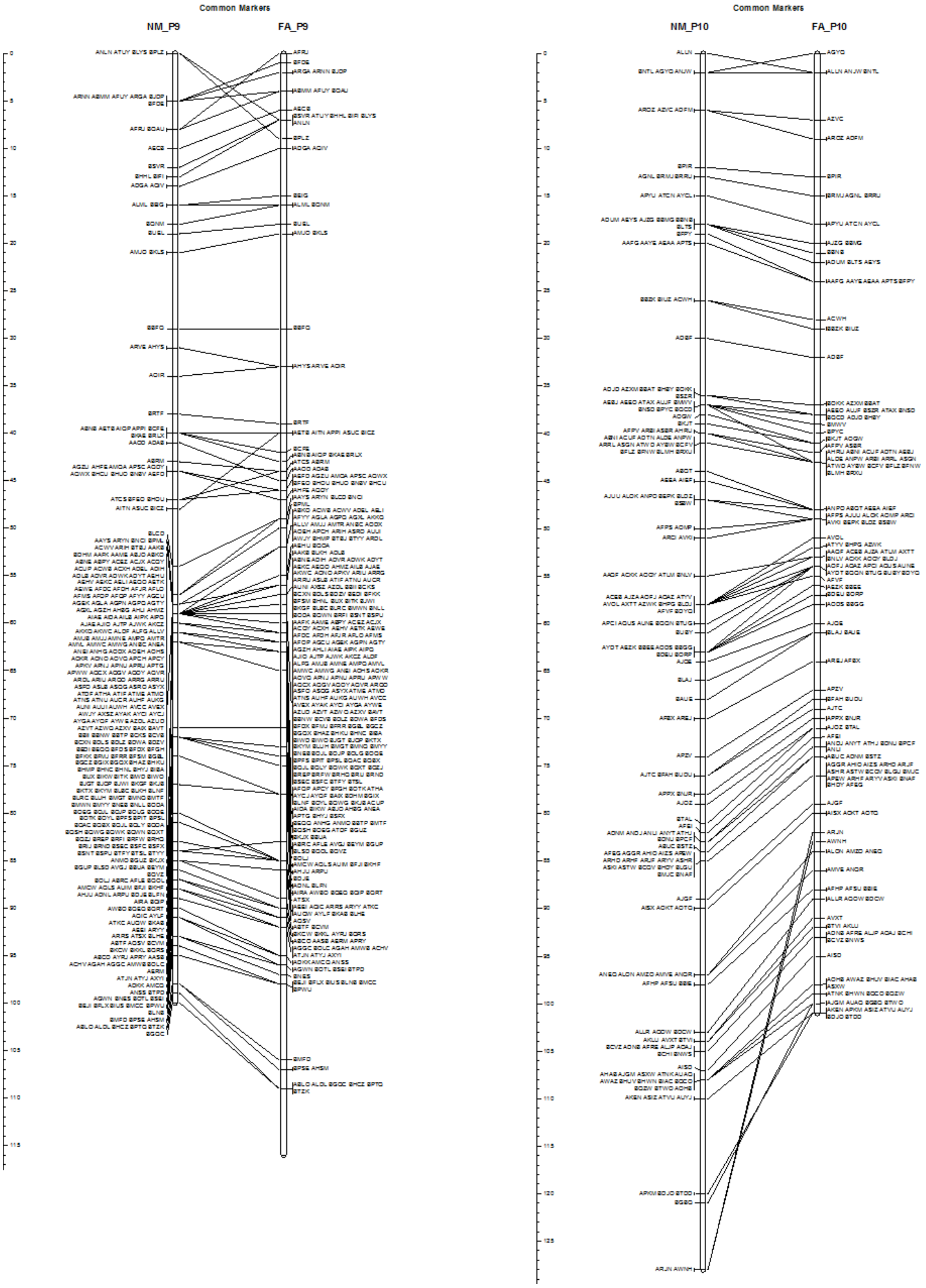
Common Markers
NM_P9
FA_P9
Common Markers
NM_P10
FA_P10

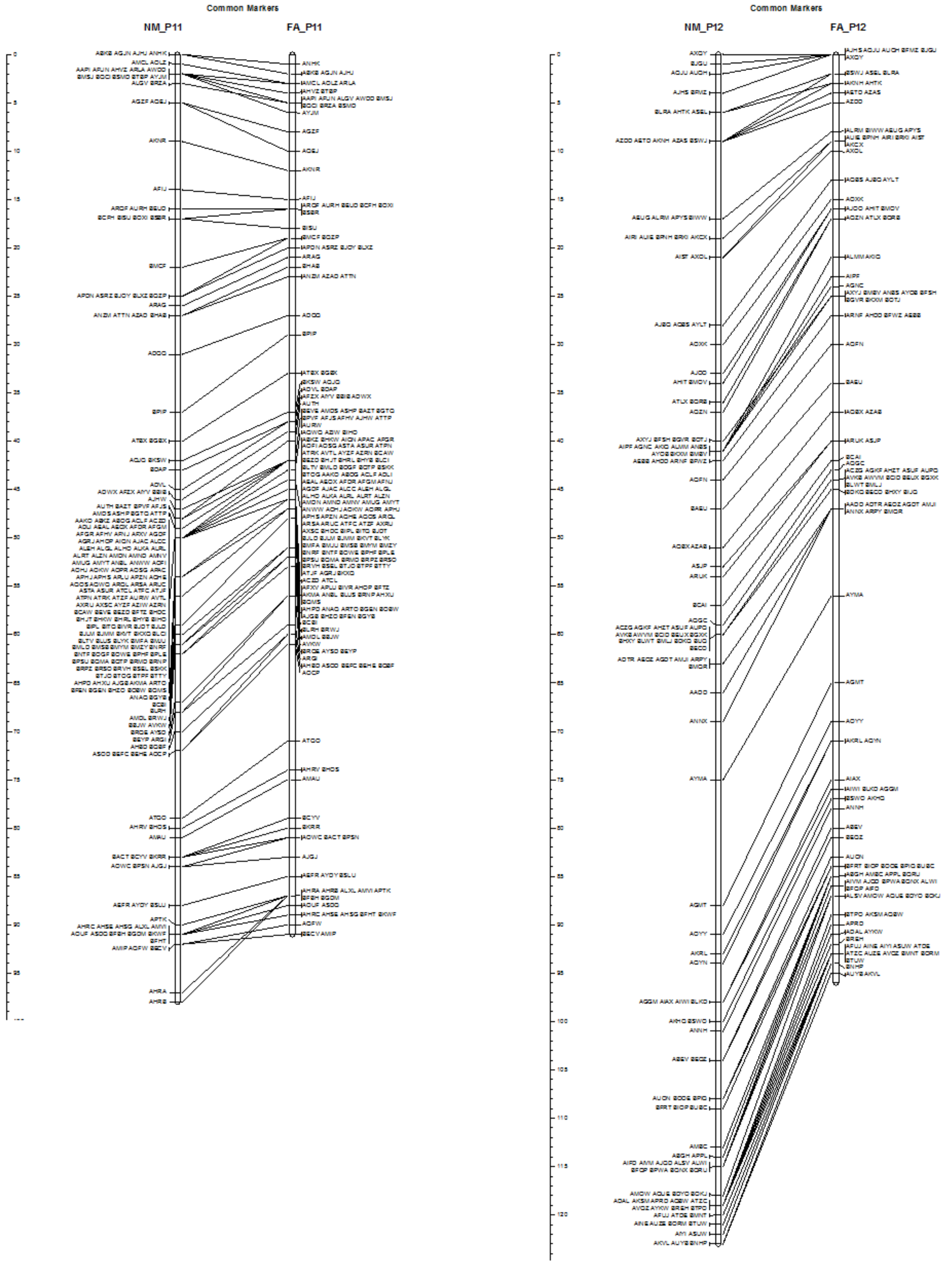

Figure S2. Comparative maps between *C. frutescens* acc. BG2814-6 × *C. annuum* 'NuMex RNaky' (FA) and *C. annuum* 'Early Jalapeño ' × *C. annuum* 'CM344' (NM) RIL populations. The map positions for 2,108 markers common to both maps found on linkage groups 2 thru 7 and 9 thru 12. Common markers between maps are connected by solid lines.

A

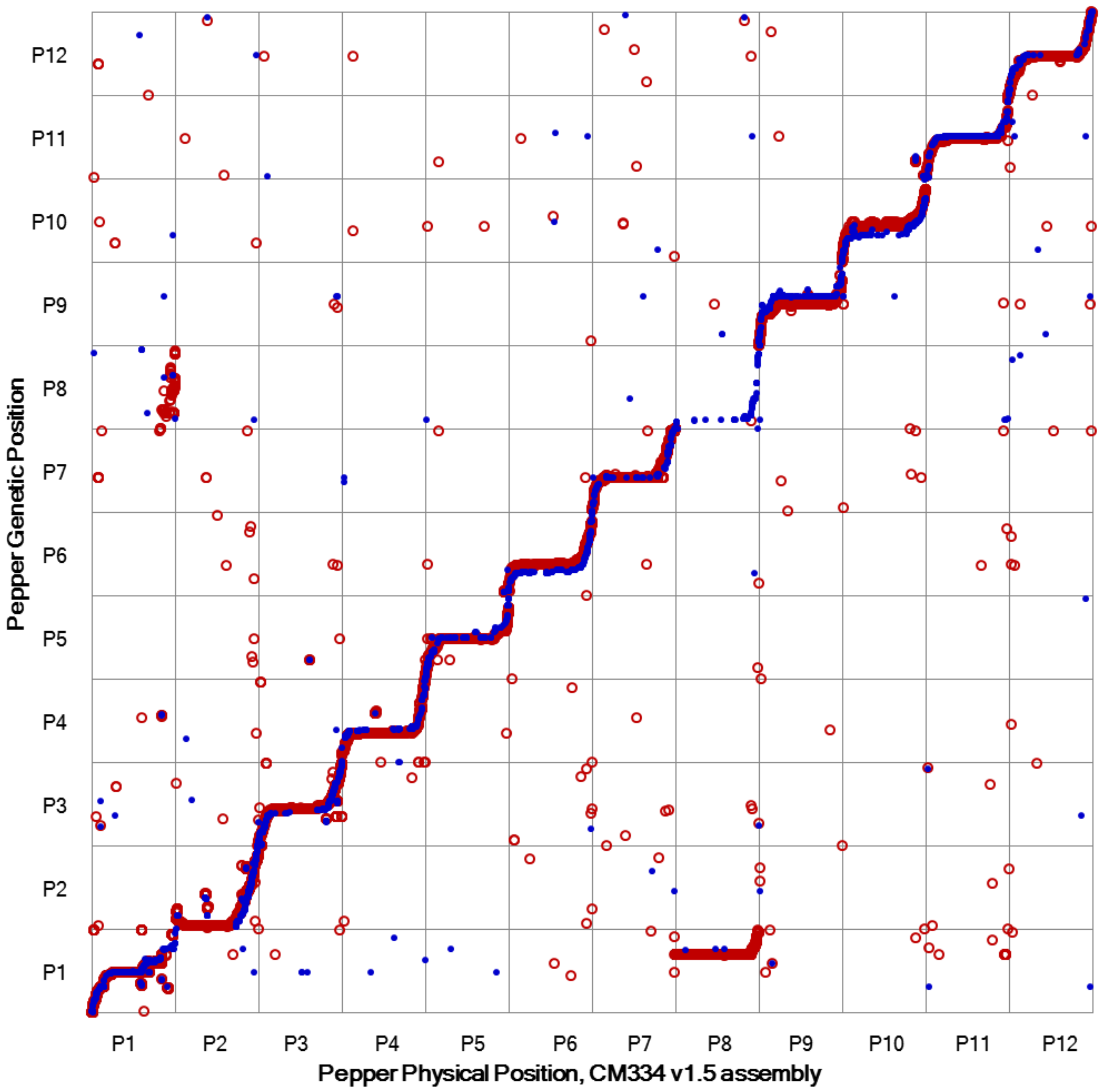

P12
P11
P10
P9
P8
P7
P6
P5
P4
P3
P2
P1
Pepper Genetic Position
P1
P2
P3
P4
P5
P6
P7
P8
P9
P10
P11
P12
Pepper Physical Position, CM334 v1.5 assembly

B

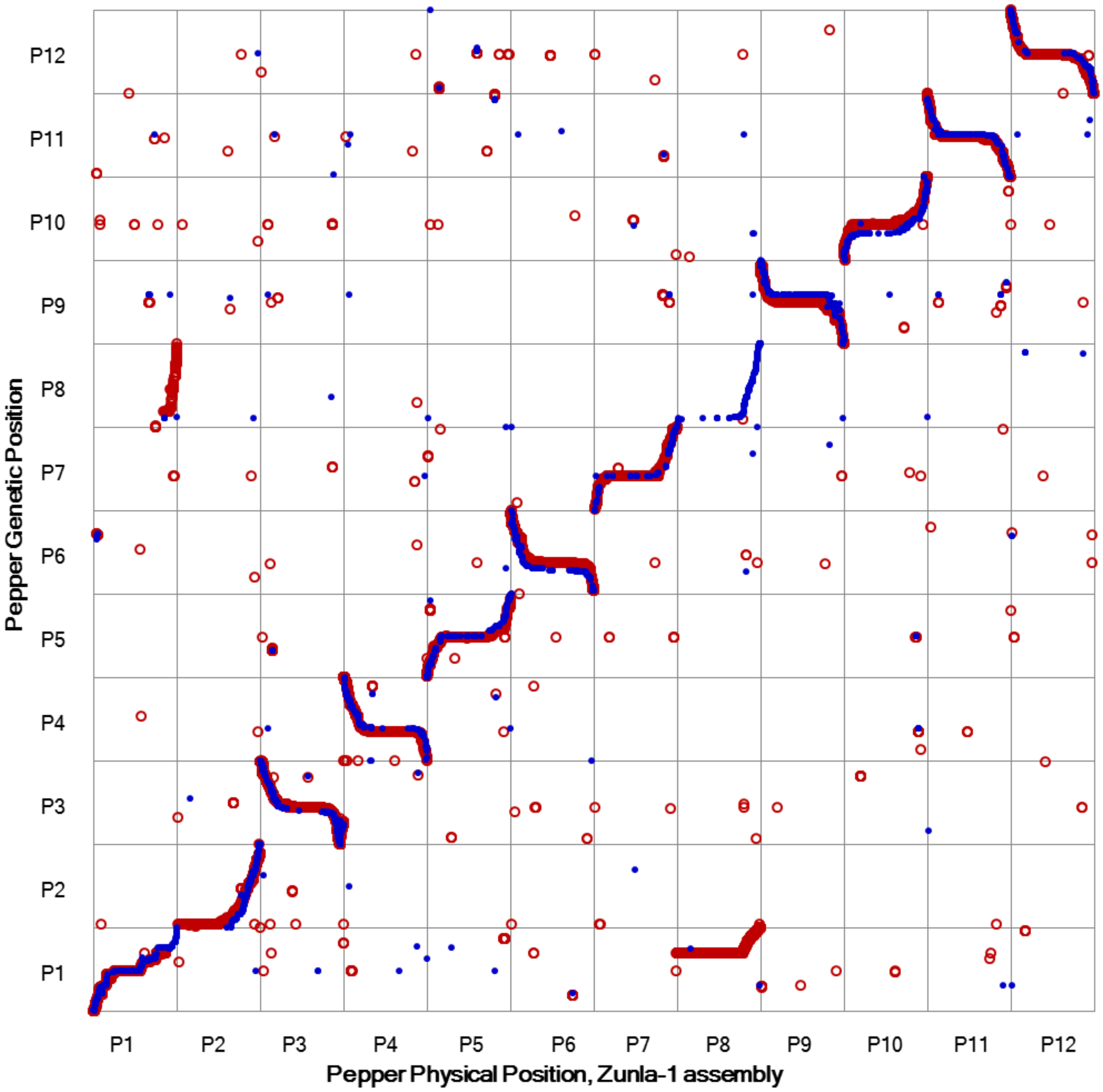

Figure S3. Pepper maps vs pepper genome assemblies. The genetic and physical postions of FA (○) and NM (●) mapped unigenes, with unique hits to chromosome pseudomolecules at ≥98% identity are shown. (A) 2621 NM and 11078 FA unigenes hitting the CM334 v1.5 genome. (B) 2779 NM and 12250 FA unigenes hitting the Zunla-1 v2.0 assembly.

A

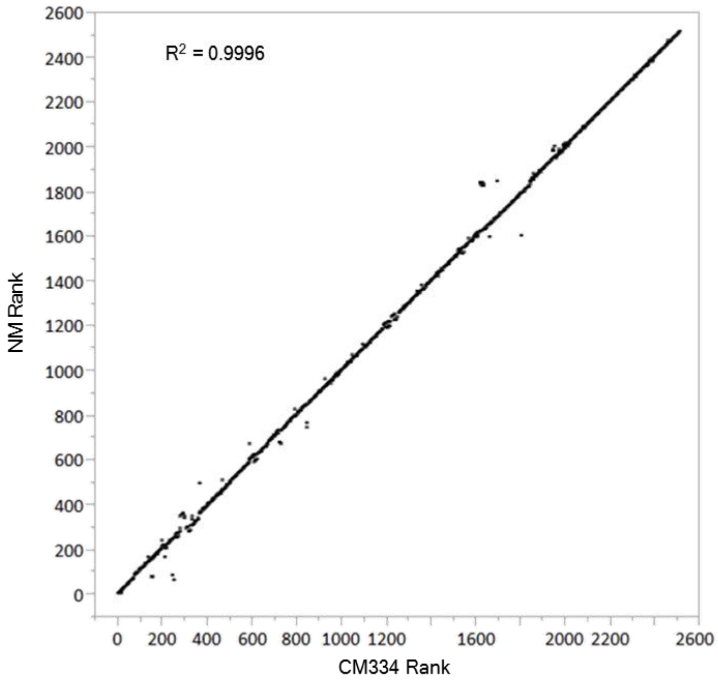
R2 = 0.9996
NM Rank
CM334 Rank
0
200
400
600
800
1000
1200
1400
1600
1800
2000
2200
2400
2600

B

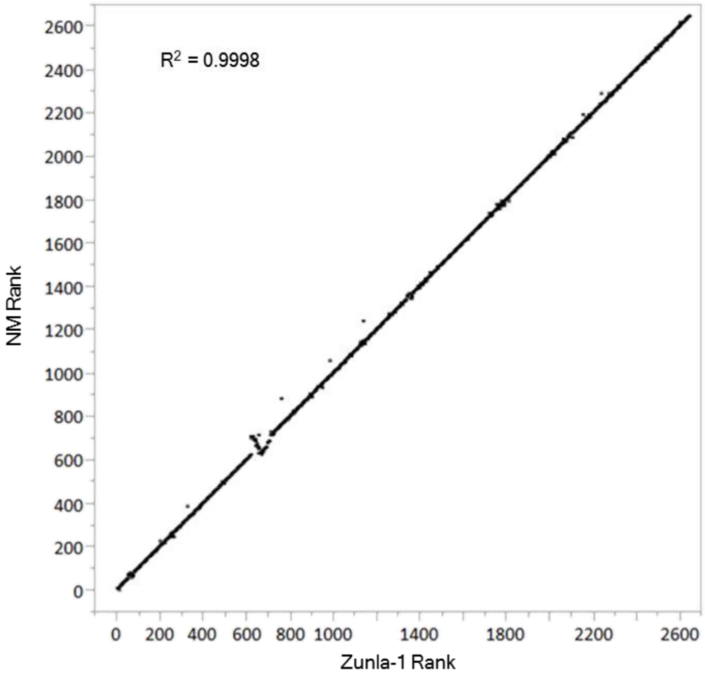
R2 = 0.9998
NM Rank
Zunla-1 Rank
0
200
400
600
800
1000
1200
1400
1600
1800
2000
2200
2400
2600

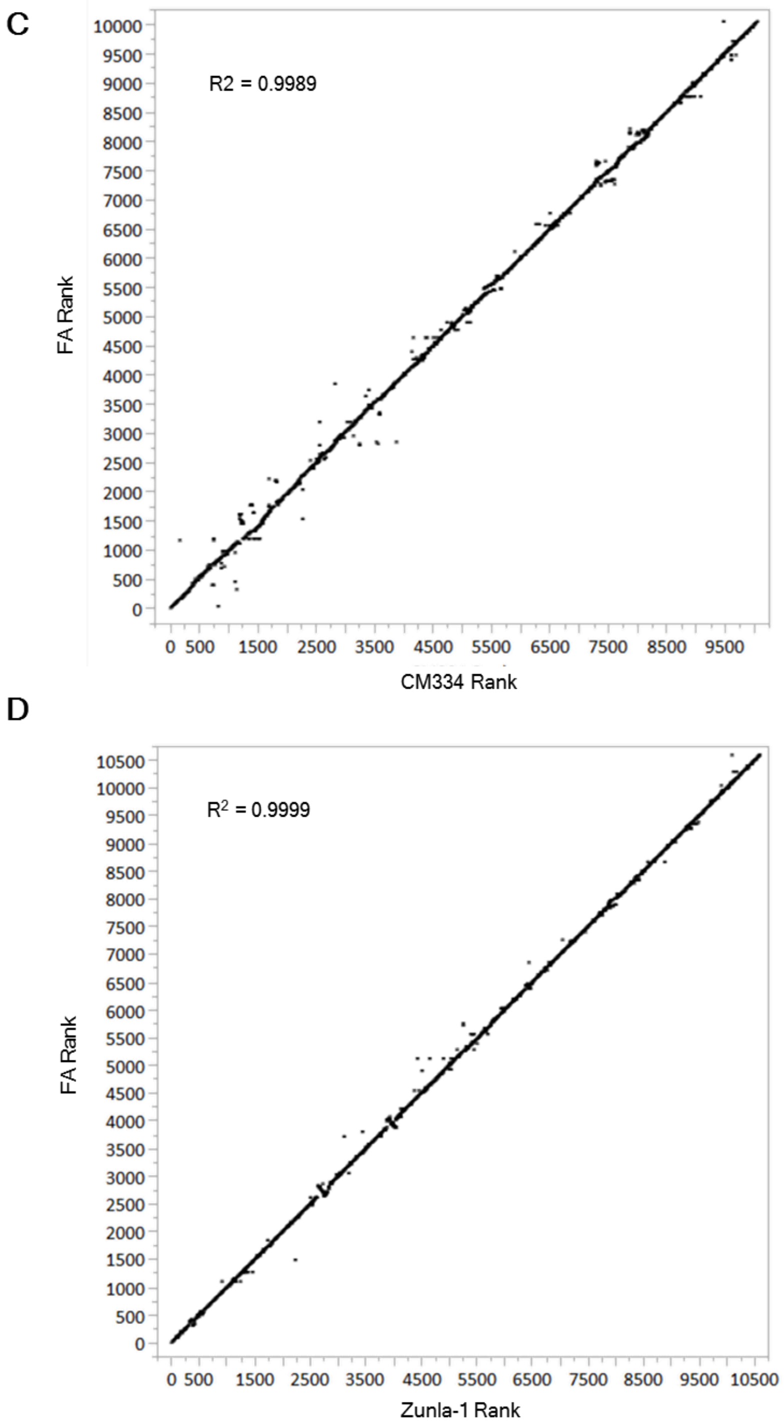

Figure S4. Regression of marker order between mapped unigenes on common linkage group/chromosome pairs. 2514 and 2653 NM unigenes found on (A) CM334 and (B) Zunla-1 chromosomes. 10033 and 10589 FA unigenes found on (C) CM334 and (D) Zunla-1 chromosome pseudomolecules. Markers were ranked based on their map order and by physical positions. Rank orders were used for regression analysis to calculate collinearity coefficients (R^2).

Files S1-S5

Available for download at www.g3journal.org/lookup/suppl/doi:10.1534/g3.115.020040/-/DC1

File S1. Data tables for *C. frutescens* acc. BG2814-6 × *C. annuum* ' NuMex RNaky' (FA07) map.

FA ReadMe. Descriptions of Tables and definitions of column names.

FA Table 1 v6. *C. frutescence* BG2814-6 X *C. annuum* NuMex RNaky Bin Map

FA Table 2 v6. Consensus haplotype for each mapped bin

FA Table 3 v6. *C. frutescence* BG2814-6 X *C. annuum* NuMex RNaky map including all mapped markers

FA Table 4 v6. Consensus haplotype for each mapped marker

Table 6. Table of conversion of four letter IDs to original contig names
Four letter codes were used for mapping to shorten IDs and allow for easy alignment on graphical map and genotype images.
FA Table 7. Cross reference of column numbers to RIL IDs in mapping files

FA Table 8 v6. Summary table of the linkage groups

FA Table 9 v6. Contigs with multiple haplotypes/markers where 2 markers were mapped

File S2. Data tables for *C. annuum* 'Early Jalapeño ' × *C. annuum* 'CM344' (NM06) map.

NM ReadMe. Descriptions of Tables and definitions of column names.

NM Table 1 v3. *Capsicum annuum* Criollo de Morelos 334 (CM334) X *C. annuum* cv Early Jalapeno bin map.

NM Table 2 v3. Consensus haplotype for each mapped bin.

NM Table 3 v3. *Capsicum annuum* Criollo de Morelos 334 (CM334) X *C. annuum* cv Early Jalapeno map including all mapped markers.

NM Table 4 v3. Consensus haplotype for each mapped marker.

Table 6. Table of conversion of four letter IDs to original contig names.
Four letter codes were used for mapping to shorten IDs and allow for easy alignment on graphical map and genotype images.

NM Table 7. Cross reference of column numbers to RIL IDs in mapping files.

NM Table 8 v3. Summary table of Bins and Markers by linkage group.

NM Table 9 v3. Contigs with multiple haplotypes/markers where 2 markers were mapped.

File S3. Common map markers. Data table for markers common to both *C. frutescens* acc. BG2814-6 X *C. annuum* ' NuMex RNaky' (FA) and *C. annuum* 'Early Jalapeño ' X C. annuum 'CM344' (NM) maps with respective map positions.

File S4. Maps vs pepper assemblies. Data tables for Pepper GeneChip unigenes with unique matches to the CM334 (v1.5) and Zunla-1 (v2.0) genomes.

Table 1 CM334 with Maps. Location of unigene contigs in the CM334 genome with map positions in *C. frutescens* acc. BG2814-6 X *C. annuum* ' NuMex RNaky' (FA) and/or *C. annuum* 'Early Jalapeño ' X C. annuum 'CM344' (NM) maps.

Table 2 Zunla-1 with Maps. Location of unigene contigs in the Zunla-1 genome with map positions in *C. frutescens* acc. BG2814-6 X *C. annuum* ' NuMex RNaky' (FA) and/or *C. annuum* 'Early Jalapeño ' X C. annuum 'CM344' (NM) maps.

Table 3 CM334 Scaffs & FA pos. CM334 Chr00 scaffolds matching FA map unigenes with corresponding FA linkage groups, positions and number of markers mapped at each position.

Table 4 CM334 Scaffs & FA by LG. CM334 Chr00 scaffolds matching FA map unigenes summarized by scaffold/linkage group pairs.

Table 5 CM334 Scaffs & NM by LG. CM334 Chr00 scaffolds matching NM map unigenes summarized by scaffold/linkage group pairs.

File S5. Maps vs tomato and potato assemblies. Data tables for Pepper GeneChip unigenes with unique matches to the tomato (SL2.50) and potato (Stuberosum_206_v3.4) genomes.

Table 1 Tomato with Maps. Location of unigene contigs in the tomato (SL2.50) genome with map positions in *C. frutescens* acc. BG2814-6 X *C. annuum* ' NuMex RNaky' (FA) and/or *C. annuum* 'Early Jalapeño ' X *C. annuum* 'CM344' (NM) maps.

Table 2 Zunla-1 with Maps. Location of unigene contigs in the potato (Stuberosum_206_v3.4) genome with map positions in *C. frutescens* acc. BG2814-6 X *C. annuum* ' NuMex RNaky' (FA) and/or *C. annuum* 'Early Jalapeño ' X *C. annuum* 'CM344' (NM) maps.
