# Supplementary material for: Ultra-High Density, Transcript-Based Genetic Maps of Pepper Define Recombination in the Genome and Synteny Among Related Species
Source: G3 (Bethesda). 2015 Sep 8;5(11):2341–55. doi: 10.1534/g3.115.020040 (PMC4632054; doi:10.1534/g3.115.020040)
Supplement: Supporting Information [file supp_g3.115.020040_TableS1.pdf]

Table S1. Summary of overall allele counts.

	# Calls	A	B	H	Missing
NM Bins	49,329	24,867 (50.4)	23,335 (47.3)	781 (1.6)	346 (0.7)
FA Bins	250,852	123,708 (49.3)	114,560 (45.7)	11,032 (4.4)	1,552 (0.6)
