# Supplementary material for: Ultra-High Density, Transcript-Based Genetic Maps of Pepper Define Recombination in the Genome and Synteny Among Related Species
Source: G3 (Bethesda). 2015 Sep 8;5(11):2341–55. doi: 10.1534/g3.115.020040 (PMC4632054; doi:10.1534/g3.115.020040)
Supplement: Supporting Information [file supp_g3.115.020040_TableS2.pdf]

Table S2. Distribution of genetic bin types

	NM map Genetic Bins	FA map Genetic Bins
Singltons	290	779
Multiple markers	493	1326
Total	783	2105
