# Supplementary material for: Ultra-High Density, Transcript-Based Genetic Maps of Pepper Define Recombination in the Genome and Synteny Among Related Species
Source: G3 (Bethesda). 2015 Sep 8;5(11):2341–55. doi: 10.1534/g3.115.020040 (PMC4632054; doi:10.1534/g3.115.020040)
Supplement: Supporting Information [file supp_g3.115.020040_TableS3.pdf]

Table S3. Size and positions of largest 1 cM bins.

Linkage Group	FA map			NM Map		
	Maximum 1 cM Bin Size	Position (cM)	% Markers	Maximum 1 cM Bin Size	Position (cM)	% Markers
P1	624	138	20%	82	84	18%
P2	286	5	17%	46	52	11%
P3	271	65	13%	34	75	8%
P4	279	46	23%	24	87	10%
P5	220	47	23%	55	46	22%
P6	214	52	15%	29	38	10%
P7	375	45	31%	25	48	17%
P8	21	13	9%	28	12	11%
P9	250	58	25%	282	60	54%
P10	238	44	22%	27	42	9%
P11	243	45	24%	177	51	49%
P12	276	45	25%	25	120	11%
