# Supplementary material for: Ultra-High Density, Transcript-Based Genetic Maps of Pepper Define Recombination in the Genome and Synteny Among Related Species
Source: G3 (Bethesda). 2015 Sep 8;5(11):2341–55. doi: 10.1534/g3.115.020040 (PMC4632054; doi:10.1534/g3.115.020040)
Supplement: Supporting Information [file supp_g3.115.020040_TableS4.pdf]

Table S4. NM regions with segregation distortion.

LG	Direction	Range (cM)	Span (cM)
1	Early Jalapeño	120-120	< 1
3	CM334	58-64	6
4	Early Jalapeño	0-5	5
5	CM334	15-23	8
	Early Jalapeño	87-91	4
6	CM334	13-32	19
	Early Jalapeño	95-104	9
7	Early Jalapeño	38-38	< 1
	Early Jalapeño	100-111	11
9	CM334	2-2	< 1
	Early Jalapeño	47-63	16
	Early Jalapeño	90-95	5
10	Early Jalapeño	54-55	1
	Early Jalapeño	128-128	< 1
11	Early Jalapeño	0-14	14
