# Supplementary material for: Ultra-High Density, Transcript-Based Genetic Maps of Pepper Define Recombination in the Genome and Synteny Among Related Species
Source: G3 (Bethesda). 2015 Sep 8;5(11):2341–55. doi: 10.1534/g3.115.020040 (PMC4632054; doi:10.1534/g3.115.020040)
Supplement: Supporting Information [file supp_g3.115.020040_TableS5.pdf]

Table S5. FA regions with segregation distortion.

LG	Direction	Range (cM)	Span (cM)
1	*C. frutescens*	0-2	2
	NuMex RNaky	22-70	48
	NuMex RNaky	104-112	8
2	*C. frutescens*	0-22*	22
	NuMex RNaky	56-59	3
	NuMex RNaky	67-72*	5
	NuMex RNaky	76-95*	19
4	NuMex RNaky	78-94*	16
	NuMex RNaky	100-131*	32
5	NuMex RNaky	20-21	1
	NuMex RNaky	65-70	5
6	NuMex RNaky	41-46*	5
	NuMex RNaky	78-83	4
	NuMex RNaky	91-95	4
7	NuMex RNaky	10-11	1
	NuMex RNaky	21-22	1
	C. frutescens	47-52	5
	NuMex RNaky	105-107	2
9	NuMex RNaky	45-78	33
	NuMex RNaky	84-107*	23
10	NuMex RNaky	40-40	<1
	NuMex RNaky	53-70	17
11	*C. frutescens*	18-18	<1
	C. frutescens	29-49	19
12	*C. frutescens*	32-58	25

*Regions that include FA QTL listed in Table S6.
