# Supplementary material for: Ultra-High Density, Transcript-Based Genetic Maps of Pepper Define Recombination in the Genome and Synteny Among Related Species
Source: G3 (Bethesda). 2015 Sep 8;5(11):2341–55. doi: 10.1534/g3.115.020040 (PMC4632054; doi:10.1534/g3.115.020040)
Supplement: Supporting Information [file supp_g3.115.020040_TableS6.pdf]

Table S6. QTL overlapping with regions of skewness in the FA population.

Direction of Skewness	FA QTL Trait	LG	Peak (cM)	LOD	R2	P value	Additive
C. frutescens	Branching Density	2	0.7	3.9	0.120	0.025	-0.31
NuMex Rnaky	Days to Breaker	2	70.4	5.1	0.174	0.005	-6.32
NuMex Rnaky	Days to Breaker	2	80.9	8.3	0.269	0.001	-8.16
NuMex Rnaky	Branching Density	4	81.8	6.4	0.182	0.001	0.28
NuMex Rnaky	Stigma Exsertion	4	113.1	3.6	0.112	0.030	-0.13
NuMex Rnaky	Days to Flowering	6	39.8	5.1	0.153	0.005	-3.84
NuMex Rnaky	Days to Flowering	9	97.5	5.9	0.180	0.001	-4.32
