# Supplementary material for: Ultra-High Density, Transcript-Based Genetic Maps of Pepper Define Recombination in the Genome and Synteny Among Related Species
Source: G3 (Bethesda). 2015 Sep 8;5(11):2341–55. doi: 10.1534/g3.115.020040 (PMC4632054; doi:10.1534/g3.115.020040)
Supplement: Supporting Information [file supp_g3.115.020040_TableS7.pdf]

Tables S7. The number of common markers for each NM and FA linkage group pair.

	NM LGs												
FA LGs	1	2	3	4	5	6	7	8	9	10	11	12	Total
1	248		1	2	1	2	1	177	2	1	2		437
2	1	265	1		1	1	1		1	1		1	273
3	2	1	274	1			1	2			2	1	284
4	1	2		173		1	1	1	1		2		182
5	2		1	1	166	1		2	1				174
6		1				199			2		1		203
7		3	1				91		1	1		1	98
8	56				1			1	1				59
9		2	1		1		1	1	364				370
10		1	1		2				1	196			201
11	1		2					1			234		238
12	2				1				2	1	2	140	148
Total	313	275	282	177	173	204	96	185	376	200	243	143	2,667
Same LG	97%	96%	97%	98%	96%	98%	95%	96%	97%	98%	96%	98%	97%
