# Supplementary material for: Ultra-High Density, Transcript-Based Genetic Maps of Pepper Define Recombination in the Genome and Synteny Among Related Species
Source: G3 (Bethesda). 2015 Sep 8;5(11):2341–55. doi: 10.1534/g3.115.020040 (PMC4632054; doi:10.1534/g3.115.020040)
Supplement: Supporting Information [file supp_g3.115.020040_TableS8.pdf]

Table S8. Summary of GMAP results for Chip assembly (unigenes) versus CM334 v1.5 and Zunla-1 v2.0 genome assemblies. The number of unigenes mapped to any linkage group identified on each chromosome pseudomolecule is also shown.

	CM334 v1.5			Zunla-1 v2.0		
Target	ESTs	NM markers	FA markers	ESTs	NM markers	FA markers
Chr01	2258	292	1334	2477	311	1429
Chr02	2348	321	1382	2347	331	1384
Chr03	2334	296	1379	2800	321	1632
Chr04	1182	116	714	1594	166	930
Chr05	1175	177	680	1333	193	762
Chr06	1498	180	843	1911	184	1078
Chr07	1622	101	947	1363	102	787
Chr08	1115	108	725	1881	195	1171
Chr09	1169	365	670	1314	361	733
Chr10	1407	222	815	1398	226	777
Chr11	1207	264	654	1242	213	667
Chr12	1615	176	926	1534	170	892
Chr 1-12	18930	2618	11069	21194	2773	12242
Chr00	6542	820	3629	2138	352	1246
Total	25472	3438	14698	23332	3125	13488
