# Supplementary material for: Ultra-High Density, Transcript-Based Genetic Maps of Pepper Define Recombination in the Genome and Synteny Among Related Species
Source: G3 (Bethesda). 2015 Sep 8;5(11):2341–55. doi: 10.1534/g3.115.020040 (PMC4632054; doi:10.1534/g3.115.020040)
Supplement: Supporting Information [file supp_g3.115.020040_TableS9.pdf]

Table S9. NM map vs CM334 v1.5 genome. The number of map markers placed on CM334 pseudomolecules for each linkage group/chromosome pair. Unigenes on the same linkage group as chromosome were used to calculate the coefficients of colinearity and recombination rates.

CM334	NM Linkage Group												
**Chr**	**1**	**2**	**3**	**4**	**5**	**6**	**7**	**8**	**9**	**10**	**11**	**12**	**Total**
1	273		3	2				11	1	1		1	292
2	2	314	1	1				1				2	321
3	3		286	1	2				3		1		296
4	2			112			2						116
5	3				164	9		1					177
6			1			176				1	2		180
7		2					95	1	1	1		1	101
8	3		1			1		99	2		1	1	108
9	1	1						1	362				365
10									3	207	12		222
11	1		1					4			258		264
12	1		1		1			2	2	1	3	165	176
Assembled	289	317	294	116	167	186	97	120	374	211	277	170	2618
Chr00	102	52	75	100	56	60	33	114	89	61	47	31	820
Total	391	369	369	216	223	246	130	234	463	272	324	201	3438
% Chr/LG Match	0.94	0.99	0.97	0.97	0.98	0.95	0.98	0.83	0.97	0.98	0.93	0.97	0.96
