# Supplementary material for: Ultra-High Density, Transcript-Based Genetic Maps of Pepper Define Recombination in the Genome and Synteny Among Related Species
Source: G3 (Bethesda). 2015 Sep 8;5(11):2341–55. doi: 10.1534/g3.115.020040 (PMC4632054; doi:10.1534/g3.115.020040)
Supplement: Supporting Information [file supp_g3.115.020040_TableS10.pdf]

Table S10. NM map vs Zunla-1 v2.0 genome. The number of map markers placed on Zunla pseudomolecules for each linkage group/chromosome pair. Unigenes on the same linkage group as chromosome were used to calculate the coefficients of colinearity.

Zulna-1	NM Linkage Group												
Chr	1	2	3	4	5	6	7	8	9	10	11	12	Total
1	293					3		7	7		1		311
2	1	326	1					1	1			1	331
3	2	1	309	1	4			1	1		2		321
4	2	1	1	158			1		1		2		166
5	3			2	171	1		2			6	8	193
6	1			1		179		1			2		184
7		1					96		3	1	1		102
8	2					1	1	187	1	2	1		195
9							1	1	359				361
10				10	5				1	210			226
11	1		1					1	9		201		213
12	1					2		6			3	158	170
Assembled	306	329	312	172	180	186	99	207	383	213	219	167	2773
Chr00	51	15	23	30	18	39	17	8	49	35	56	11	352
Total	357	344	335	202	198	225	116	215	432	248	275	178	3125
% Chr/LG Match	0.96	0.99	0.99	0.92	0.95	0.96	0.97	0.90	0.94	0.99	0.92	0.95	0.95
