# Supplementary material for: Ultra-High Density, Transcript-Based Genetic Maps of Pepper Define Recombination in the Genome and Synteny Among Related Species
Source: G3 (Bethesda). 2015 Sep 8;5(11):2341–55. doi: 10.1534/g3.115.020040 (PMC4632054; doi:10.1534/g3.115.020040)
Supplement: Supporting Information [file supp_g3.115.020040_TableS11.pdf]

Table S11. FA map vs CM334 v1.5 genome. The number of map markers placed on CM334 pseudomolecules for each linkage group/chromosome pair. Unigenes on the same linkage group as chromosome were used to calculate the coefficients of colinearity and recombination rates.

CM334	FA linkage group												
Chr	1	2	3	4	5	6	7	8	9	10	11	12	Total
1	1182	1	4	6			8	125		3	1	4	1331
2	1	1363	2	1	3	5	3			1	2	1	1383
3	2		1365	3	4	2			2			1	1381
4		1	1	710						1		1	717
5				1	660	15	1			2	1		685
6	2	3	5	2		827	1		1	1	1		849
7	4	1	5	1		1	928			3	1	3	951
8	716		3		1	1		1	1			2	731
9	3	2		2			2		659		1	1	678
10	1	1	1				5		2	787	18		825
11	15	2	2			2	1		1		631		665
12	1	1	1	1		3	2		2	2	1	912	26
Assembled	1927	1375	1389	727	668	856	951	126	668	800	657	925	11069
Chr00	954	184	458	386	208	481	153	91	200	176	231	107	3629
Total	2881	1559	1847	1113	876	1337	1104	217	868	976	888	1032	14698
% Chr/LG Match	0.98	0.99	0.98	0.98	0.99	0.97	0.98	1.00	0.99	0.98	0.96	0.99	0.98
