# Supplementary material for: Ultra-High Density, Transcript-Based Genetic Maps of Pepper Define Recombination in the Genome and Synteny Among Related Species
Source: G3 (Bethesda). 2015 Sep 8;5(11):2341–55. doi: 10.1534/g3.115.020040 (PMC4632054; doi:10.1534/g3.115.020040)
Supplement: Supporting Information [file supp_g3.115.020040_TableS12.pdf]

Table S12. FA map vs Zunla-1 v2.0 genome. The number of map markers placed on Zunla-1 pseudomolecules for each linkage group/chromosome pair. Unigenes on the same linkage group as chromosome were used to calculate the coefficients of colinearity.

Zunla-1	FA linkage group												
Chr	1	2	3	4	5	6	7	8	9	10	11	12	Total
1	1256	1		1		9	3	141	6	5	6	1	1429
2	1	1366	9	1		1	1		1	2	1	1	1384
3	5	6	1585		13	1	4		4	12	1	1	1632
4	11	1	1	910		1	2	1			2	1	930
5	14		3	2	687	1	5			2	20	28	762
6	13	1	6	1	1	1046	1			1		8	1078
7	2	2	2		6	1	752		10	4	5	3	787
8	1161	1	3			3		1		1		1	1171
9	10		1			1	2		718			1	733
10	3		13	11	4		2		2	742			777
11	2	1		3		1	1		11	2	646		667
12	5		3		4	3	1		1	3		872	892
Assembled	2483	1379	1626	929	715	1068	774	143	753	774	681	917	12242
Chr00	154	70	102	112	87	176	194	35	67	103	110	36	1246
Total	2637	1449	1728	1041	802	1244	968	178	820	877	791	953	13488
% Chr/LG Match	0.97	0.99	0.97	0.98	0.96	0.98	0.97	0.99	0.95	0.96	0.95	0.95	0.97
