# Supplementary material for: Ultra-High Density, Transcript-Based Genetic Maps of Pepper Define Recombination in the Genome and Synteny Among Related Species
Source: G3 (Bethesda). 2015 Sep 8;5(11):2341–55. doi: 10.1534/g3.115.020040 (PMC4632054; doi:10.1534/g3.115.020040)
Supplement: Supporting Information [file supp_g3.115.020040_TableS13.pdf]

Table S13. NM map vs Tomato v2.5 genome. The number of map markers placed on tomato pseudomolecules (Sl 2.5) for each linkage group/chromosome pair.

Tomato	NM Linkage Group												
Chr	1	2	3	4	5	6	7	8	9	10	11	12	Total
1	103		1	1	1	1	1	117	2	2	1		230
2	2	196	4			1	2	1	7	3		1	217
3	4		105	42	2	1	1		3	1		2	161
4	3	2	1	57	57				2	3	2	9	136
5		1	2	7	46	1			2	1	57	1	118
6	1	2			1	106	1	2	1	1	2	3	120
7		2	2	2		2	56		2			2	68
8	70	1						23	4		3		101
9	1	3	75				2	3	139		3	1	227
10	2	1	1		2	2		1	3	114	1		127
11	2			9	1			2	1	1	80	37	133
12	4	1	4	1	3		5		49	5	3	37	112
Total	192	209	195	119	113	114	68	149	215	131	152	93	1,750
