# Supplementary material for: Ultra-High Density, Transcript-Based Genetic Maps of Pepper Define Recombination in the Genome and Synteny Among Related Species
Source: G3 (Bethesda). 2015 Sep 8;5(11):2341–55. doi: 10.1534/g3.115.020040 (PMC4632054; doi:10.1534/g3.115.020040)
Supplement: Supporting Information [file supp_g3.115.020040_TableS14.pdf]

Table S14. NM map vs Potato v 2.06 genome. The number of map markers placed on potato pseudomolecules for each linkage group/chromosome pair.

Potato	NM Linkage Group												
Chr	1	2	3	4	5	6	7	8	9	10	11	12	Total
1	103	1	2	1	1	3	1	111	3	2	1		229
2	2	217	4			2	1	1	6	1		1	235
3	4	1	101	38	1		1		1			3	150
4	2	2		60	53				3		2	9	131
5		1	1	8	45				2		55	1	113
6	1	2			1	99	3	2	1		2	2	113
7	2	3	3	2		2	56		2			1	71
8	74							27	4		3		108
9	2	3	64		1			4	132		2	1	209
10	3		3			2			3	119		1	131
11	1		1	9		1		2	1	1	73	37	126
12	3	1	3	1	2	1			52	2	2	36	103
Total	197	231	182	119	104	110	62	147	210	125	140	92	1,719
