# Supplementary material for: Ultra-High Density, Transcript-Based Genetic Maps of Pepper Define Recombination in the Genome and Synteny Among Related Species
Source: G3 (Bethesda). 2015 Sep 8;5(11):2341–55. doi: 10.1534/g3.115.020040 (PMC4632054; doi:10.1534/g3.115.020040)
Supplement: Supporting Information [file supp_g3.115.020040_TableS15.pdf]

Table S15. FA map vs Tomato v2.5 genome. The number of map markers placed on tomato (Sl 2.5) pseudomolecules for each linkage group/chromosome pair.

Tomato	FA Linkage Group												
Chr	1	2	3	4	5	6	7	8	9	10	11	12	Total
1	1,051	4	7	6	2	2	1	5	2	2		4	1,086
2	5	820	6	4		2	7		3	4	3	3	857
3	15	5	684	95	1	4			8	3	1	9	825
4	9	2	2	384	196	3	4	2	2	7	7	82	700
5	5	6	1	11	184	3	4		3	1	233	8	459
6	16	4	5	4	1	612	4	1	5	2	5	3	662
7	9	14	7	4	4	4	551		8	2	1	7	611
8	437	4	2	6	2	4	3	78	3	2	4	3	548
9	8	5	239	4	3	4	3		260	6	2	3	537
10	12	5	4	2	4	2	3	1	3	434	1	1	472
11	5	4	3	69	5	4	1		3	5	191	173	463
12	4	3	57	20	3		3	1	129	6	5	201	432
Total	1576	876	1017	609	405	644	584	88	429	474	453	497	7,652
