# Supplementary material for: Ultra-High Density, Transcript-Based Genetic Maps of Pepper Define Recombination in the Genome and Synteny Among Related Species
Source: G3 (Bethesda). 2015 Sep 8;5(11):2341–55. doi: 10.1534/g3.115.020040 (PMC4632054; doi:10.1534/g3.115.020040)
Supplement: Supporting Information [file supp_g3.115.020040_TableS16.pdf]

Table S16. FA map vs Potato v 2.06 genome. The number of map markers placed on potato pseudomolecules for each linkage group/chromosome pair.

Potato	FA Linkage Group												
Chr	1	2	3	4	5	6	7	8	9	10	11	12	Total
1	1,071	4	6	3	4	3	1	2		6	1	6	1,107
2	6	838	6	7	1	2	6		2	3	2	3	876
3	15	10	657	77	3	4			6	2	2	4	780
4	8	3	2	372	181	3	3	3	1	4	5	82	667
5	5	6	2	12	184	4	5		5	2	225	7	457
6	20	3	5	3	1	606	5		4	4	1	2	654
7	10	10	8	4	3	4	534		5	3	2	9	592
8	433	2	1	8	1	4	3	78	3	3			536
9	13	6	231	6	3	4	3		244	3	1	3	517
10	8	3	5	5	1	2	3		3	427		1	458
11	5	3	4	67	3	8	3		2	4	178	165	442
12	2	4	75	17	4	2	2	1	132	3	5	210	457
Total	1,596	892	1,002	581	389	646	568	84	407	464	422	492	7,543
