# Supplementary material for: Ultra-High Density, Transcript-Based Genetic Maps of Pepper Define Recombination in the Genome and Synteny Among Related Species
Source: G3 (Bethesda). 2015 Sep 8;5(11):2341–55. doi: 10.1534/g3.115.020040 (PMC4632054; doi:10.1534/g3.115.020040)
Supplement: Supporting Information [file supp_g3.115.020040_FigureS1.pdf]

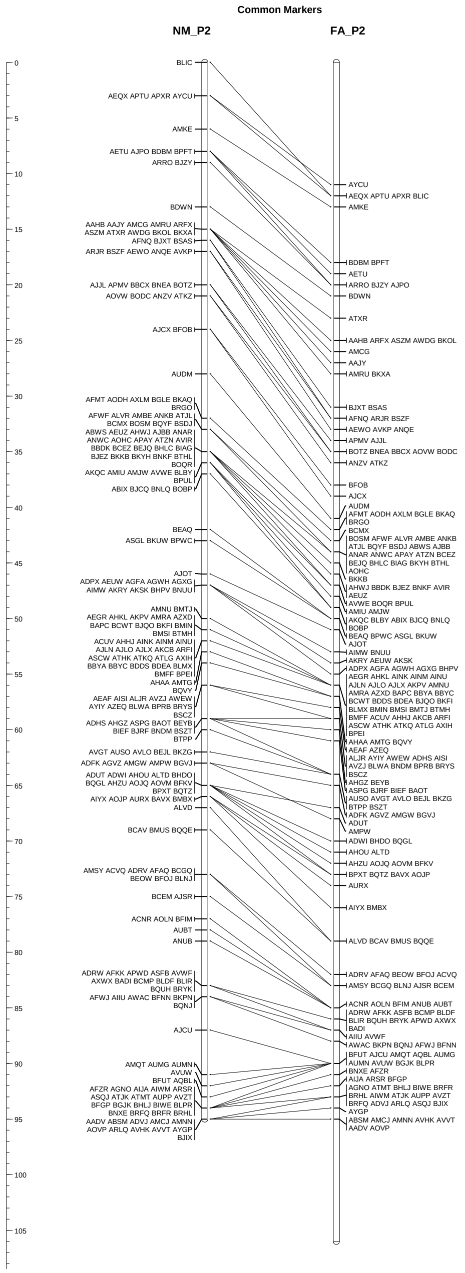
Common Markers
NM_P2
FA_P2

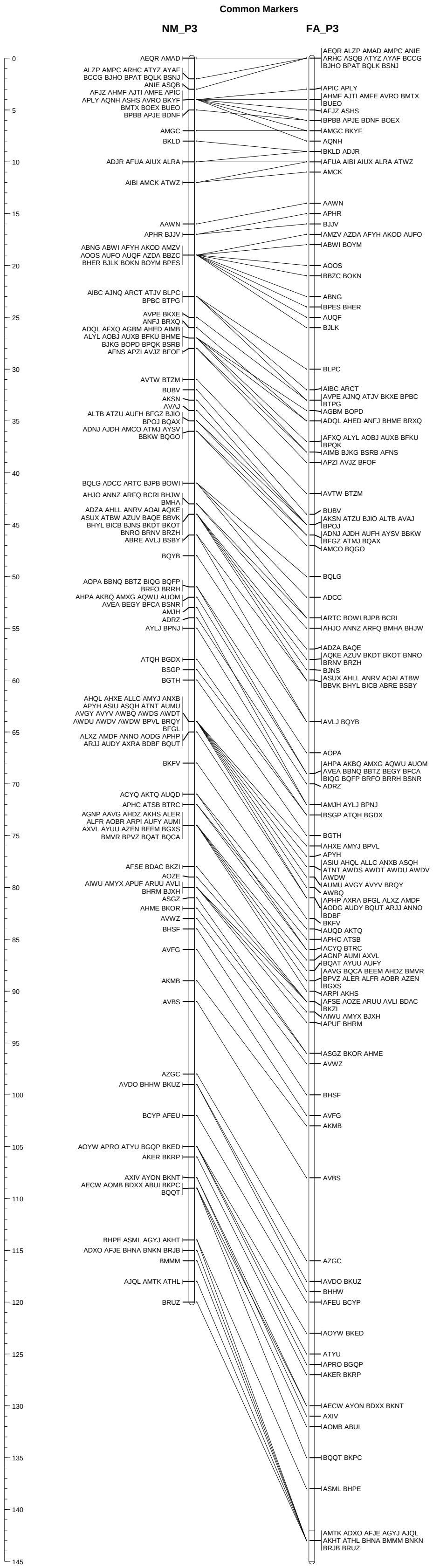
Common Markers
NM_P3
FA_P3

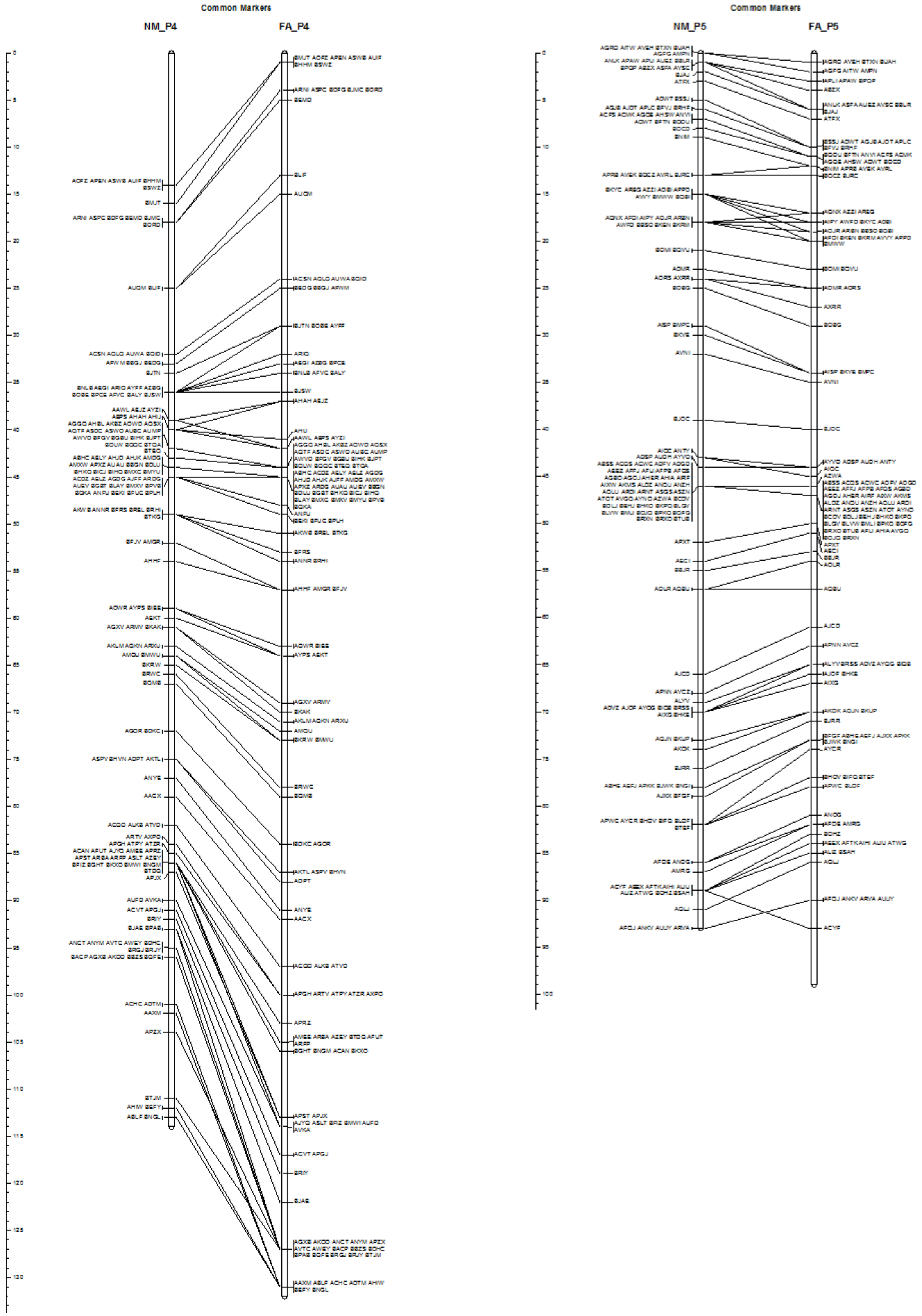
Common Markers
NM_P4
FA_P4
Common Markers
NM_P5
FA_P5

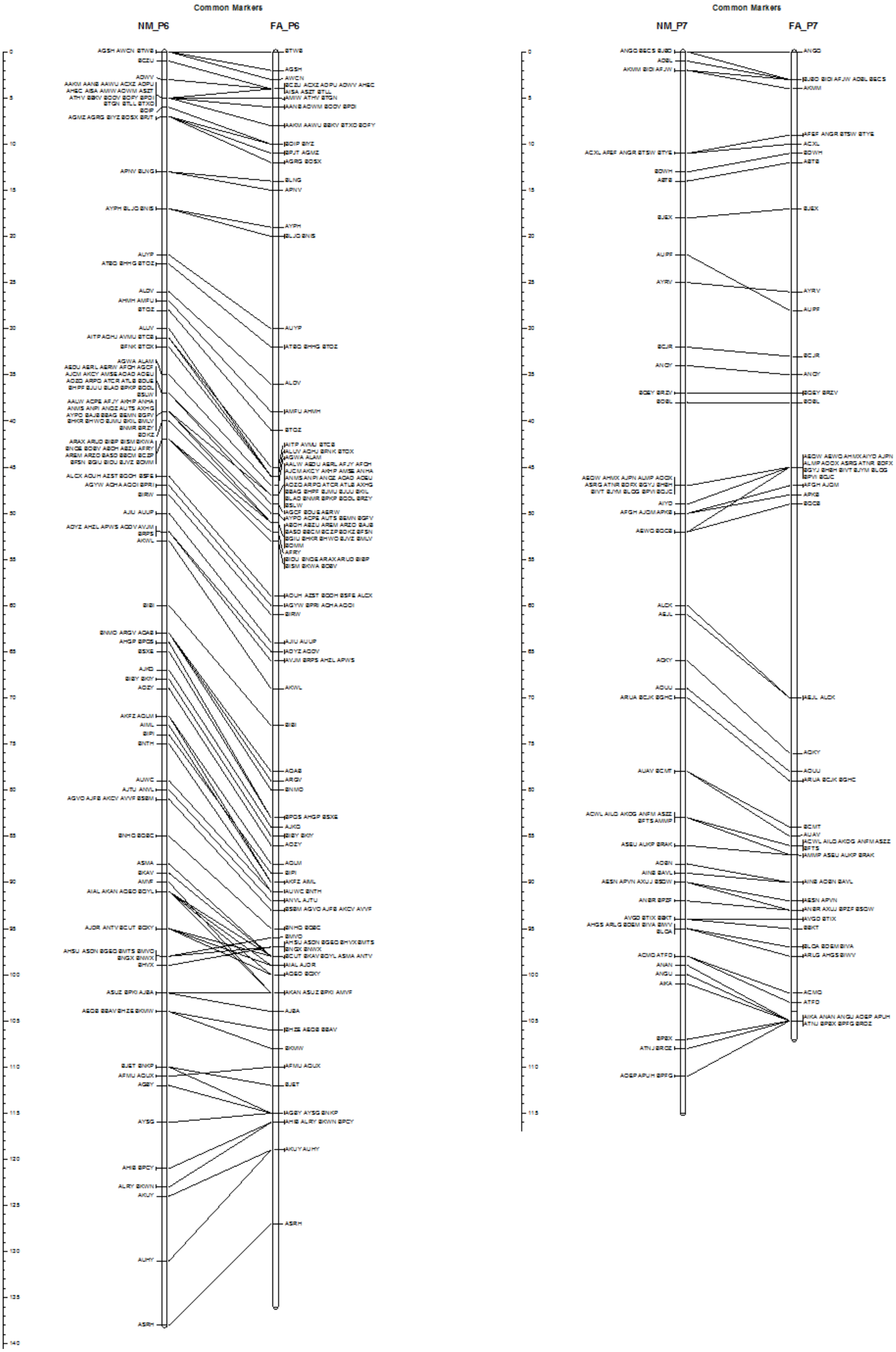
Common Markers
NM_P6
FA_P6
Common Markers
NM_P7
FA_P7

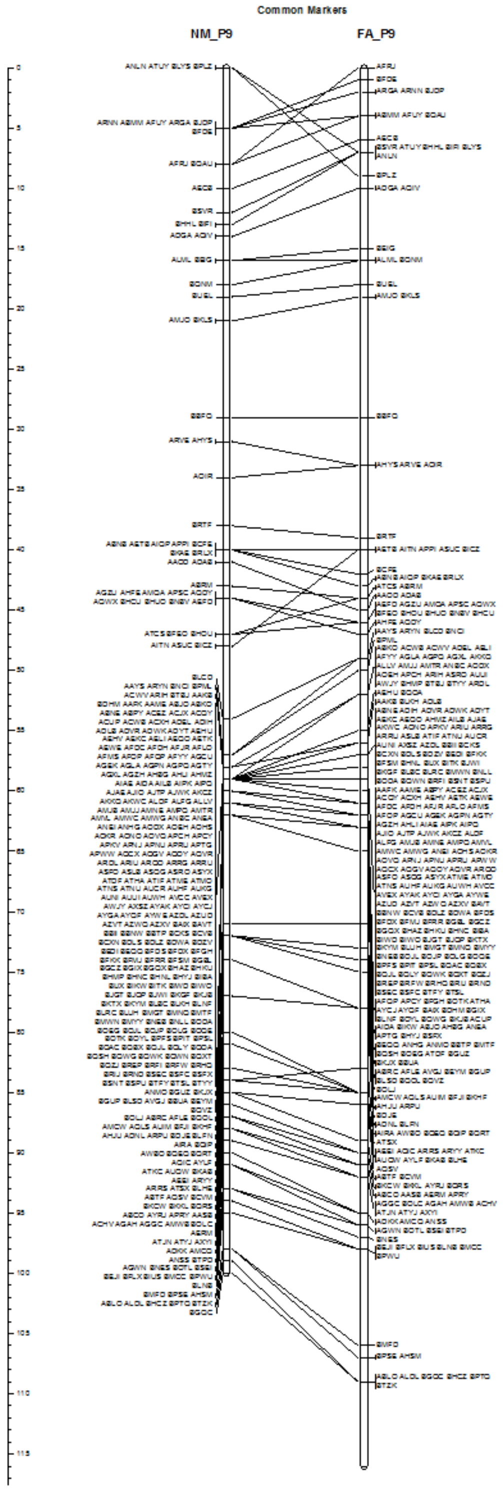
Common Markers
NM_P9
FA_P9

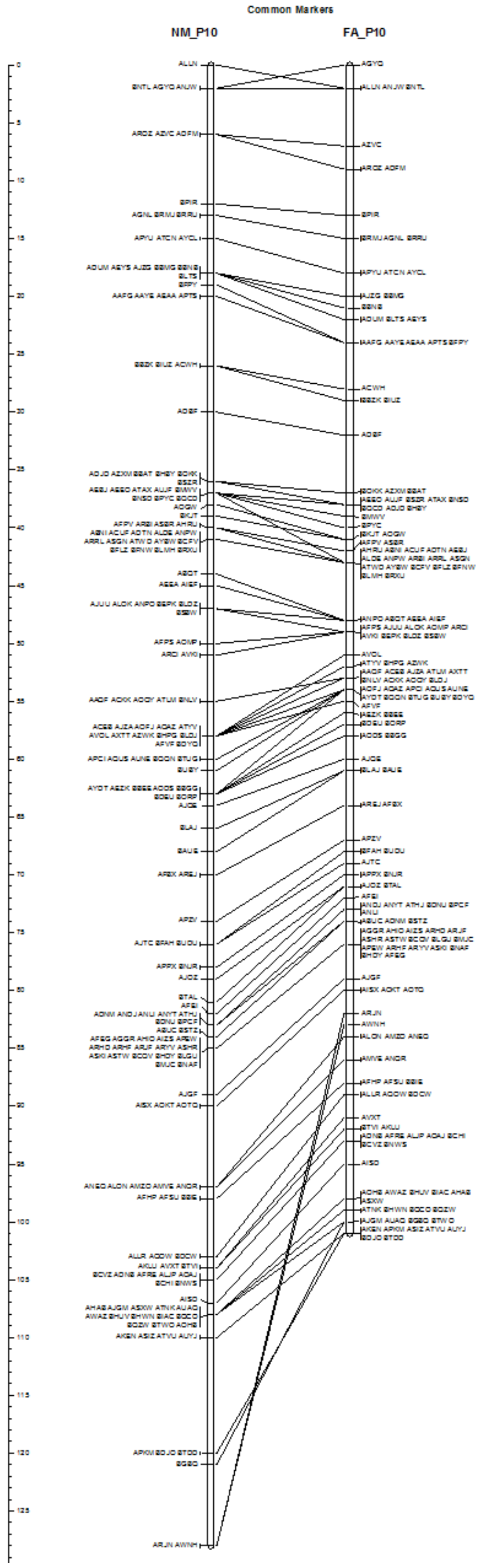
Common Markers
NM_P10
FA_P10

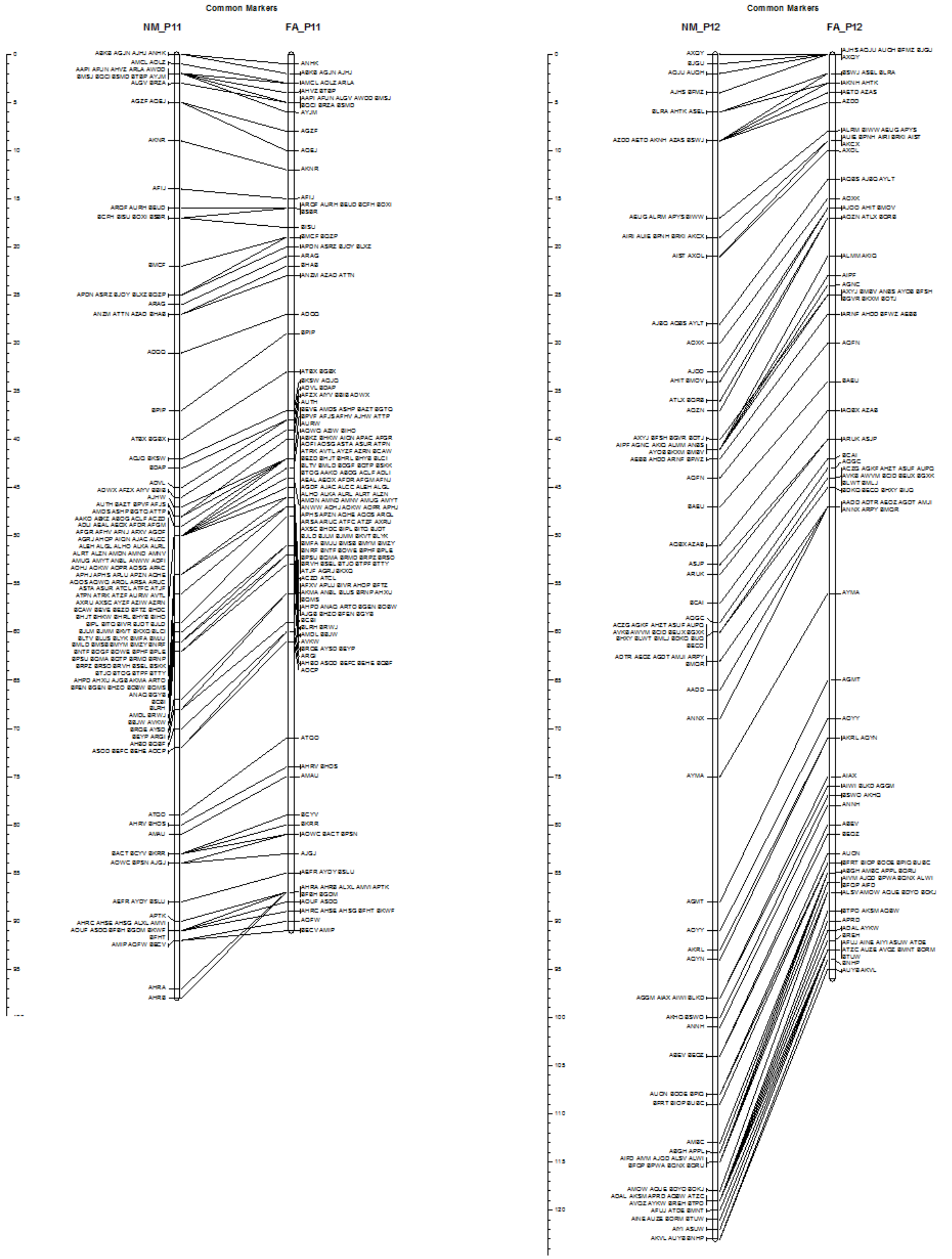

Figure S2. Comparative maps between *C. frutescens* acc. BG2814-6 × *C. annuum* ‘NuMex RNaky’ (FA) and *C. annuum* ‘Early Jalapeño ’ × *C. annuum* ‘CM344’ (NM) RIL populations. The map positions for 2,108 markers common to both maps found on linkage groups 2 thru 7 and 9 thru 12. Common markers between maps are connected by solid lines.
