# Supplementary material for: Ultra-High Density, Transcript-Based Genetic Maps of Pepper Define Recombination in the Genome and Synteny Among Related Species
Source: G3 (Bethesda). 2015 Sep 8;5(11):2341–55. doi: 10.1534/g3.115.020040 (PMC4632054; doi:10.1534/g3.115.020040)
Supplement: Supporting Information [file supp_g3.115.020040_FigureS3.pdf]

A

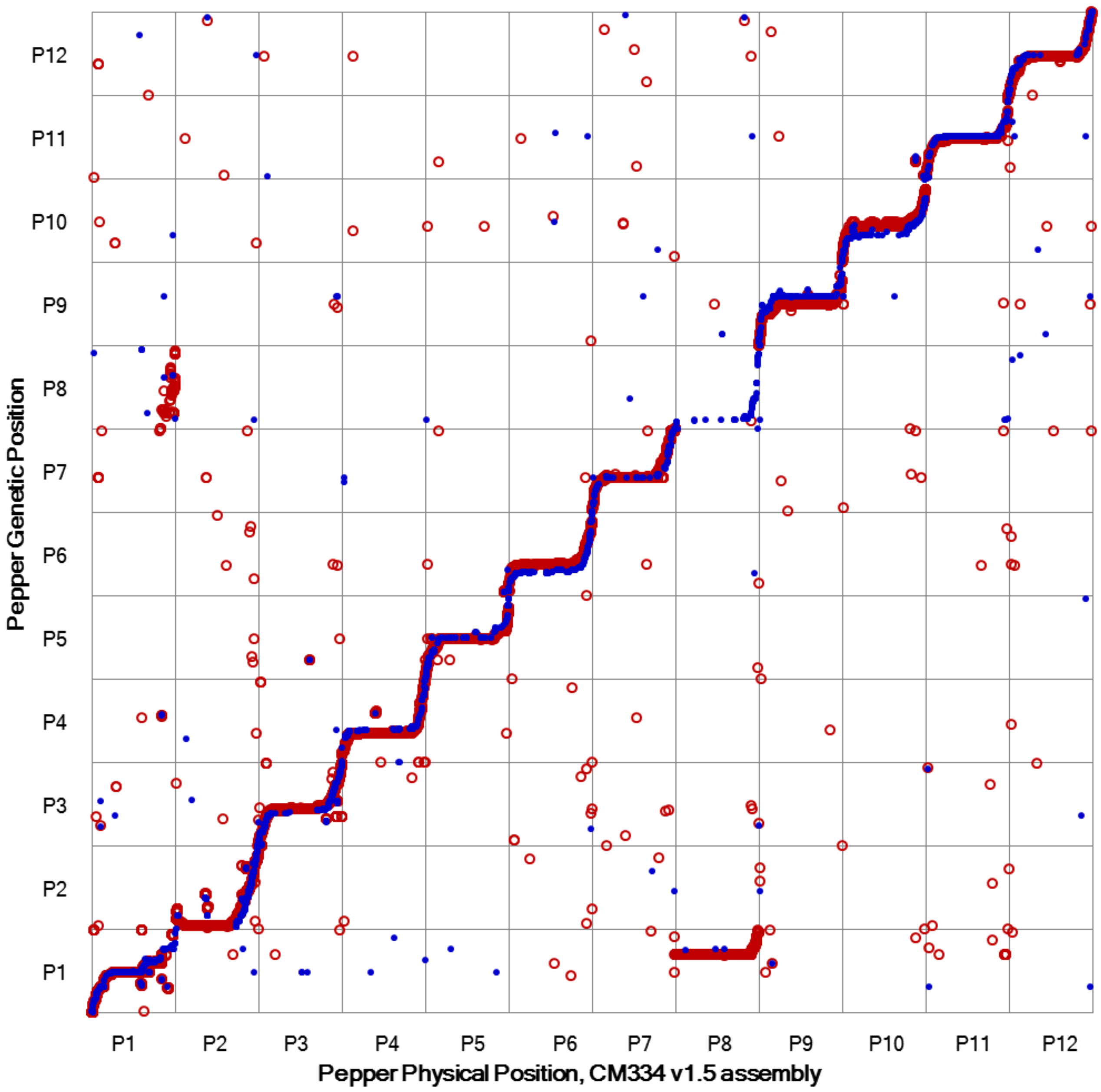

P12
P11
P10
P9
P8
P7
P6
P5
P4
P3
P2
P1
Pepper Genetic Position
P1
P2
P3
P4
P5
P6
P7
P8
P9
P10
P11
P12
Pepper Physical Position, CM334 v1.5 assembly

B

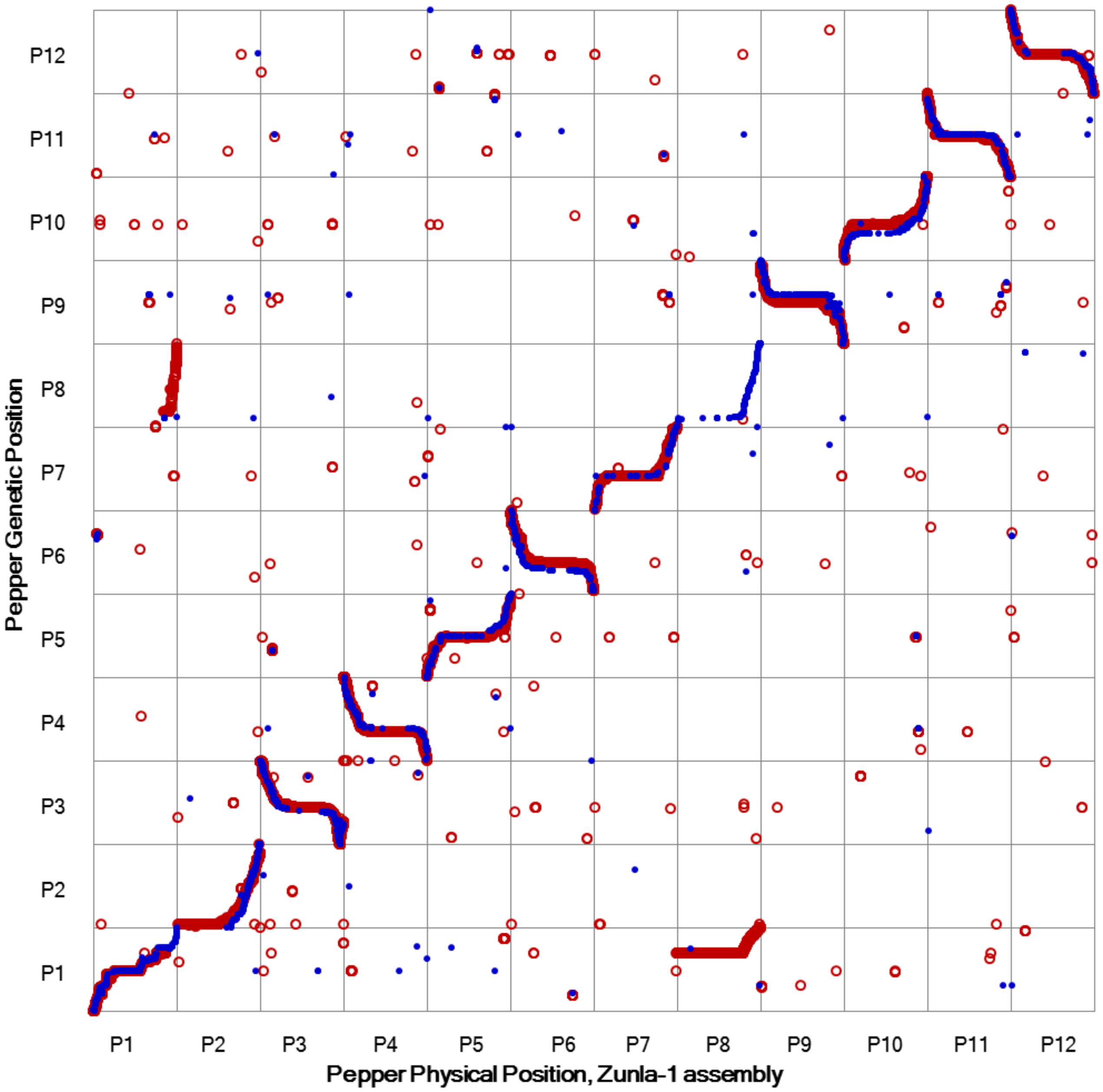

Figure S3. Pepper maps vs pepper genome assemblies. The genetic and physical postions of FA (○) and NM (●) mapped unigenes, with unique hits to chromosome pseudomolecules at ≥98% identity are shown. (A) 2621 NM and 11078 FA unigenes hitting the CM334 v1.5 genome. (B) 2779 NM and 12250 FA unigenes hitting the Zunla-1 v2.0 assembly.
