# Supplementary material for: Ultra-High Density, Transcript-Based Genetic Maps of Pepper Define Recombination in the Genome and Synteny Among Related Species
Source: G3 (Bethesda). 2015 Sep 8;5(11):2341–55. doi: 10.1534/g3.115.020040 (PMC4632054; doi:10.1534/g3.115.020040)
Supplement: Supporting Information [file supp_g3.115.020040_FigureS4.pdf]

A

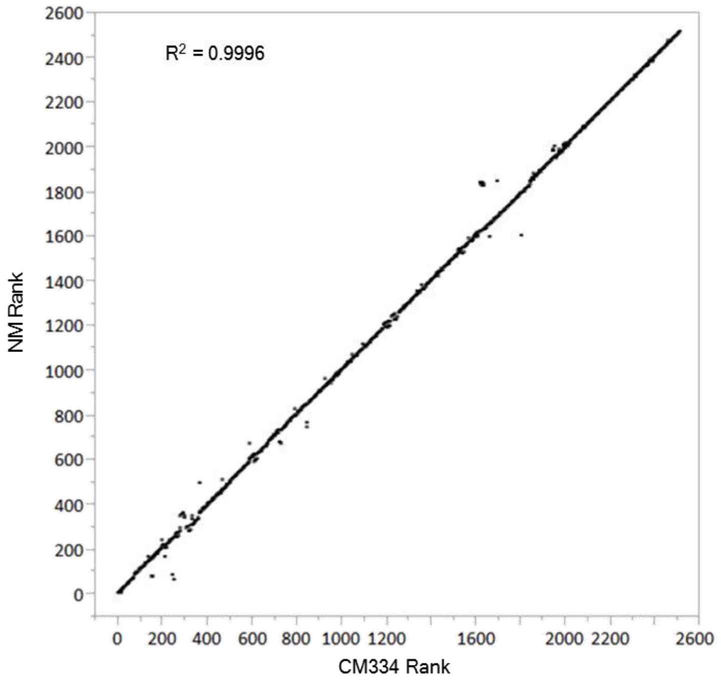

R² = 0.9996
NM Rank
CM334 Rank
0
200
400
600
800
1000
1200
1400
1600
1800
2000
2200
2400
2600

B

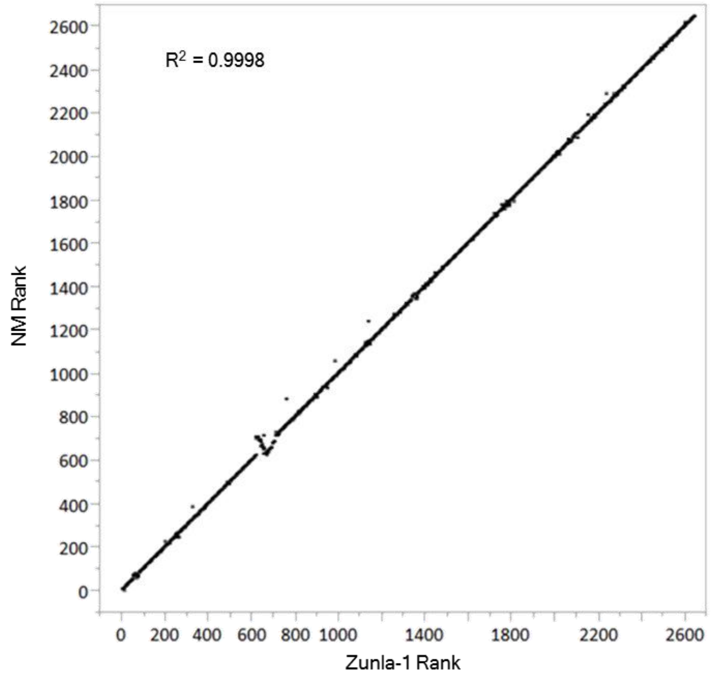

R² = 0.9998
NM Rank
Zunla-1 Rank
0
200
400
600
800
1000
1200
1400
1600
1800
2000
2200
2400
2600

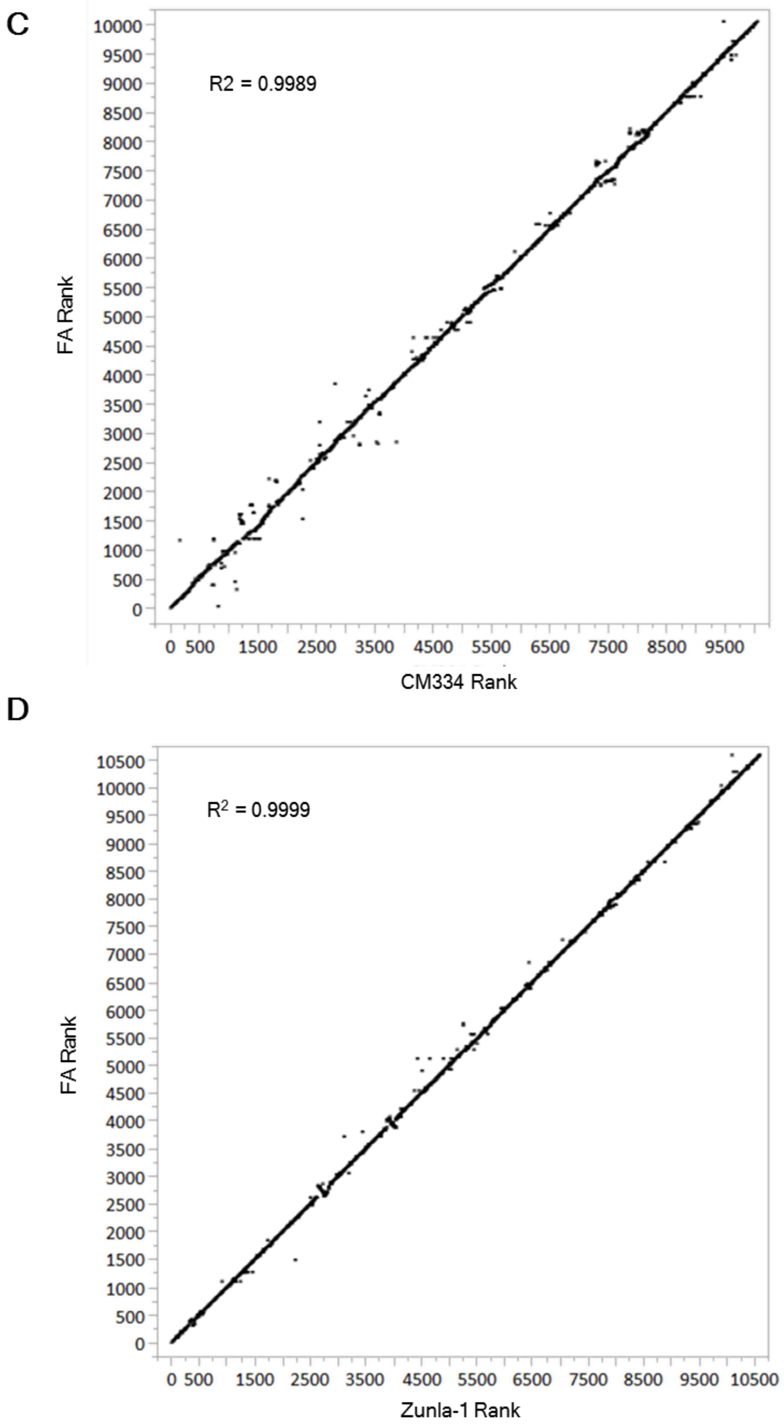

Figure S4. Regression of marker order between mapped unigenes on common linkage group/chromosome pairs. 2514 and 2653 NM unigenes found on (A) CM334 and (B) Zunla-1 chromosomes. 10033 and 10589 FA unigenes found on (C) CM334 and (D) Zunla-1 chromosome pseudomolecules. Markers were ranked based on their map order and by physical positions. Rank orders were used for regression analysis to calculate collinearity coefficients (R^2).
